# An integrated approach to correction for off-resonance effects and subject movement in diffusion MR imaging

**DOI:** 10.1016/j.neuroimage.2015.10.019

**Published:** 2016-01-15

**Authors:** Jesper L.R. Andersson, Stamatios N. Sotiropoulos

**Affiliations:** FMRIB Centre, Oxford, United Kingdom

**Keywords:** Diffusion, Eddy current, Movement, Susceptibility, Registration

## Abstract

In this paper we describe a method for retrospective estimation and correction of eddy current (EC)-induced distortions and subject movement in diffusion imaging. In addition a susceptibility-induced field can be supplied and will be incorporated into the calculations in a way that accurately reflects that the two fields (susceptibility- and EC-induced) behave differently in the presence of subject movement. The method is based on registering the individual volumes to a model free prediction of what each volume should look like, thereby enabling its use on high *b*-value data where the contrast is vastly different in different volumes. In addition we show that the linear EC-model commonly used is insufficient for the data used in the present paper (high spatial and angular resolution data acquired with Stejskal–Tanner gradients on a 3 T Siemens Verio, a 3 T Siemens Connectome Skyra or a 7 T Siemens Magnetome scanner) and that a higher order model performs significantly better.

The method is already in extensive practical use and is used by four major projects (the WU-UMinn HCP, the MGH HCP, the UK Biobank and the Whitehall studies) to correct for distortions and subject movement.

## Highlights

• We present a new method for correction of eddy current-induced distortions and subject movement in diffusion data.
• It is based on alignment to predictions based on a Gaussian process
• The results indicate that one can achieve reliable corrections even for high *b*-value data (*b* = 7000).
• A comparison to correlation ratio based registration (eddy_correct) indicates that the new method is vastly superior.

## Introduction

MR diffusion imaging is a powerful tool for examining the connectivity and microstructure of the brain (see for example Jones (2011) or Johansen-Berg and Behrens (2014) for recent summaries of the field). It is typically performed using spin echo (SE) echo-planar imaging (EPI) that has been sensitized to diffusion by additional gradients intended to spoil the signal from randomly moving water. Each scan provides information about diffusivity along one direction (specified by the diffusion gradient). A protocol typically consists of many scans that together provide information about diffusion along “any” direction (see for example Le Bihan (2011) for an introduction to diffusion imaging). Recent advances in multi-band imaging (Moeller et al., 2010, Setsompop et al., 2012 or Uğurbil et al., 2013) mean that data of unprecedented quality can be acquired with several hundred diffusion directions and multiple shells/*b*-values (S.N. Sotiropoulos et al., 2013 or Setsompop et al., 2013). This in turn means that new exciting analysis methods (Assaf et al., 2008, Sotiropoulos et al., 2012, McNab et al., 2013a) are becoming increasingly feasible, which is likely to aid biological and medical research.

On the other hand the technique is marred by artifacts such as off-resonance induced distortions and subject movement (Andersson and Skare, 2011, Pierpaoli, 2011).

EPI images are sensitive to off-resonance fields because of the low bandwidth in the phase-encode (PE) direction (Schmitt et al., 1998), which leads to telltale unidirectional distortions. One source of off-resonance is the object itself, which when placed in the scanner will disrupt the existing (homogeneous) field, rendering the resulting field inhomogeneous. This is known as a susceptibility induced field because it is caused by the different tissues in the object having different magnetic susceptibilities (*i.e.*, being differentially easy to magnetize) from each other and from the air surrounding the object.

For diffusion weighted EPI images (DWI) the strong, rapidly switched, diffusion encoding gradients are an additional source of off-resonance. The rapidly changing magnetic field induces eddy currents (EC) in conductors within the bore, which will in turn induce a magnetic field. This is known as an eddy current-induced off-resonance field.

Finally there are inevitable subject movements. This is especially true when collecting multi-shell and/or high angular resolution data (Descoteaux et al., 2006 or Alexander et al., 2006) and/or high spatial resolution data (S.N. Sotiropoulos et al., 2013 or Vu et al., 2015), where subjects are kept in the scanner for an extended period of time.

Current methods for retrospective correction often perform poorly when attempting to correct high resolution, high *b*-value data acquired with strong and fast switching gradients for diffusion weighting. They suffer either from using an inadequate spatial model for the EC-induced field (*i.e.*, the linear model proposed by Jezzard et al. (1998)) or from using a non-optimal similarity function to drive the registration. The linear model is predicated on an assumption that the eddy currents reside in the gradient coils themselves and that those currents result in a field that is a linear combination of linear gradients in the three primary directions. In this work we find, as did Rohde et al. (2004), that a linear model does not adequately describe the fields/distortions. This is hardly surprising as modern gradient systems are not particularly linear (see *e.g.*, Janke et al., 2004).

The issue of similarity function stems from the different diffusion weighted images having different contrast, which means that the common assumption (in image registration) that the images are identical except for a geometric transform is not true. This becomes an issue in particular for high *b*-value scanning where, to the eye, it can sometimes seem inconceivable that two images acquired with different diffusion gradient directions come from the same subject.

In this paper we describe a method for estimating and correcting for EC-induced distortions and subject movements. It works by making a prediction about how each diffusion weighted volume “should look” and by comparing that prediction to the observed data. The resulting “error-signal” is used to update the estimate of the EC-induced field, which can be of higher order, and the subject position (movement). The prediction is calculated using a Gaussian Process (GP) for which the hyperparameters are estimated directly from the data. See Rasmussen and Williams (2006) for a general overview of GPs and Andersson and Sotiropoulos (2015) for details of the specific GP used for this work.

If there is an existing estimate of the susceptibility-induced off-resonance field it can be incorporated into the estimation. This then provides additional information about the EC distortions and movements. It also ensures that data is only resampled once when correcting for susceptibility- and EC-induced distortions and for subject movement.

The method has previously been described briefly in Andersson et al. (2012) and S.N. Sotiropoulos et al. (2013).

## Theory

**Fig. 1:**
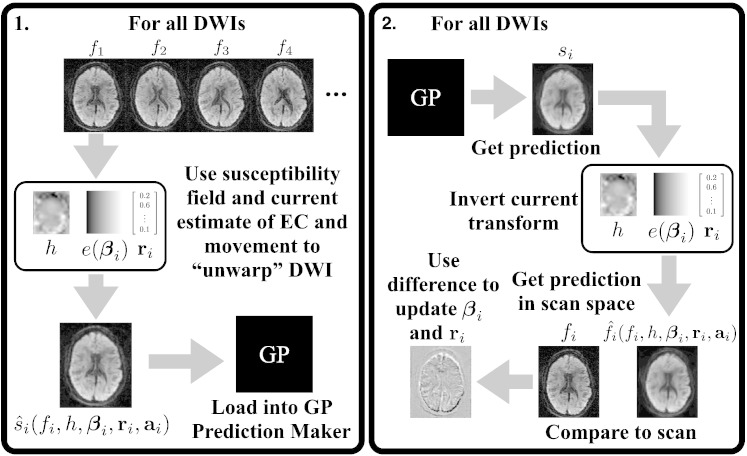
This figure explains the correction algorithm. Each iteration consists of two parts: 1) The prediction step and 2) The estimation step. It is run for a fixed number of iterations *M* and experience has shown that *M* = 5 iterations is sufficient.

In this section we will describe the proposed method in some detail. The casual reader may want to skip straight to The registration algorithm section and Fig. 1 and return to this section later for details.

### Definition of terms

(1) $$\begin{array}{lll} s & : & \mathrm{Reference}/\mathrm{undistorted}\;\mathrm{space}.\;\mathrm{Used}\;to\;\mathrm{denote}\;\mathrm{the}\;\mathrm{space}\;\mathrm{or}\;\mathrm{any}\;\mathrm{image}\;\mathrm{in}\;\mathrm{that}\;\mathrm{space}.\\ f & : & \mathrm{Observed}/\mathrm{distorted}\;\mathrm{image}.\;\mathrm{Used}\;to\;\mathrm{denote}\;\mathrm{any}\;\mathrm{image}\;\mathrm{in}\;\mathrm{acquisition}\;\mathrm{space}.\\ a & : & \mathrm{Acquisition}\;\mathrm{parameters}.\;PE‐\mathrm{direction}\;\mathrm{and}\;\mathrm{bandwidth}\;\mathrm{in}\;PE‐\mathrm{direction}.\\ r & : & \mathrm{Rigid}\;\mathrm{body}\;\left(\mathrm{subject}\;\mathrm{movement}\right)\;\mathrm{parameters}.\\ \beta & : & \mathrm{Eddy}\;\mathrm{current}\;\mathrm{parameters}.\;\mathrm{Four}\;for\;\mathrm{linear},\;\mathrm{ten}\;for\;\mathrm{quadratic}\;etc.\\ e\left(\beta \right) & : & \mathrm{Eddy}\;\mathrm{current}‐\mathrm{induced}\;\mathrm{off}\;\mathrm{resonance}\;\mathrm{field}\;\left(\mathrm{Hz}\right).\\ h & : & \mathrm{Susceptibility}\;\mathrm{induced}\;\mathrm{off}‐\mathrm{resonance}\;\mathrm{field}\;\left(\mathrm{Hz}\right).\\ \omega \left(\psi ,r\right) & : & \mathrm{Field}\;\left(\mathrm{Hz}\right)\;\mathrm{obtained}\;by\;\mathrm{rigid}\;\mathrm{body}\;\mathrm{transform}\;\mathrm{of}\;\mathrm{field}\;\psi \;\mathrm{with}\;r.\\ d\left(\psi ,a\right) & : & \mathrm{Voxel}\;\mathrm{displacement}\;\mathrm{field}\;\mathrm{given}\;\mathrm{field}\;\psi \;\left(\mathrm{Hz}\right)\;\mathrm{and}\;a\text{.} \end{array}$$

A more detailed explanation of these terms can be found in Appendix A and for readers who wish to follow the details of the algorithm is recommended reading.

In addition we will refer to “scanner space”, denoting a space that is fixed w.r.t. to the scanner coordinate system, and “subject space” as a space that is fixed w.r.t. to the (head of the) subject.

### Spatial transforms

#### Including susceptibility-induced field

The susceptibility induced field is not estimated as part of this process, but if one is provided it is used. In the majority of the theory section we will assume that one is provided. In principle, it could have been derived from a dual echo-time fieldmap sequence (Jezzard and Balaban, 1995) or from any Reverse Gradient Method (RGM) (see for example Andersson et al., 2003 or Morgan et al., 2004). In practice, the software implementing the method presented in this paper (eddy) assumes the fieldmap format given by the topup tool in FSL (http://fsl.fmrib.ox.ac.uk/fsl/fslwiki/TOPUP). If a fieldmap is not supplied, eddy will still be able to correct for eddy currents and subject movement, though the resulting space will in that case still have the susceptibility distortions.

#### Spatial model for eddy current-induced field

It is often assumed that EC-induced distortions consist of in-plane shears and zooms and translations along the PE-direction (see *e.g.*, Haselgrove and Moore, 1996, Bastin, 1999, Bastin and Armitage, 2000, Bodammer et al., 2004, Zhuang et al., 2006). This is explained in Jezzard et al. (1998) and is predicated on the assumption that the EC-induced fields can be adequately described by a linear combination of linear gradients in the principle directions and an offset. This in turn assumes that the conductors responsible for the EC-induced fields are the gradient coils themselves because there is no reason to believe that a current in any other conductor in the bore would result in a linear gradient. In contrast others have found empirically that a low order polynomial representation of the field yields better results than the linear model (Rohde et al., 2004).

Like Rohde et al. (2004) we have found that a low (second or third) order polynomial model for the field gives better results. Therefore in the present work, there are options to model the EC-induced field as a first, second or third order polynomial. The polynomial EC-induced field is combined with the susceptibility induced field in the way described in Combining the fields section and used together with the pertinent acquisition parameters to transform the distorted images as described in Resampling the images section.

It should be noted that only an off-resonance field that remains unchanged during the duration of the image encoding will cause a “simple” geometric distortion. If the eddy currents, and hence the field, changes during the encoding they will instead cause blurring along the PE direction (Xu et al., 2013). While working on the HCP acquisition protocol we saw no evidence of temporally varying EC fields being a major issue, so in this work we do not attempt to model/correct for those.

#### Combining the fields

If the subject remains absolutely motionless between the assessment of the susceptibility induced field and the acquisition of the diffusion weighted image, it would be trivial to combine the two. One would just have to add the fields together (as a very good first order approximation). Unfortunately one will always have to consider the possibility that the subject moves, so a slightly more complicated method is necessary. The two fields (the EC-induced and the susceptibility-induced) will behave differently in the presence of subject movement. The EC-field is stationary w.r.t. to the scanner space, whereas the susceptibility-induced field is to a first approximation (however, see Andersson et al. (2001) for more details) stationary w.r.t. the subject space. Let us assume that the susceptibility field *h* was estimated in the reference space *s* and that for a diffusion weighted image *f*_*i*_ the eddy current and movement parameters are given by ***β***_*i*_ and **r**_*i*_ respectively. Then the transform that takes *f*_*i*_ into *s* is given by

(2) $$x^{\prime }=R_{i}^{-1}x+d_{x}\left(h+\omega \left(e\left(\beta_{i}\right),r_{i}\right),a_{i}\right)$$

where **x** is a coordinate in *s* and **x**′ in the observed space (*f*_*i*_) and where we have abused notation slightly by using **R**_*i*_^− 1^ to denote the mapping given by Eq. (A1).

As part of the algorithm we will also need to transform predictions from the space of *s* to the space *f*_*i*_ of an acquisition and that transform is given by

(3) $$x^{\prime }=R_{i}\left(x+d_{x}{\left(\omega \left(h,r_{i}\right)+e\left(\beta_{i}\right),a_{i}\right)}^{-1}\right)$$

where **R**_*i*_ denote the inverse of the transform given by Eq. (A1) and where **d**_**x**_(*ω*(*h*, **r**_*i*_) + *e*(***β***_*i*_), **a**_*i*_)^− 1^ denotes the value at voxel **x** in the field obtained by inverting the displacement field **d**(*ω*(*h*, **r**_*i*_) + *e*(***β***_*i*_), **a**_*i*_), where the inversion is an operation on the entire field.

#### Resampling the images

When resampling the images, the intensity for a location **x**′ that does not fall on a voxel center is interpolated using cubic splines as described in Unser et al., 1993a, Unser et al., 1993b. In addition to that, one also needs to compensate for the signal stretching/compression that occurs when voxels in one space get mapped to locations closer/further apart in the other space. When signal from many voxels gets displaced into fewer voxels (by the distortions) there will be a pile-up of signal into a hyper-intense area, and likewise when signal from a few voxels gets displaced into many voxels there will be a “thinning” of the signal. This is taken into account by multiplying the signal in the resampled image by the local Jacobian of the transform. Hence, the intensity in a given voxel after resampling the observed image *f*_*i*_ into the space of *s* is given by

(4) $${\hat{s}}_{i}\left(x;f_{i},h,\beta_{i},r_{i},a_{i}\right)=f_{i}\left(x^{\prime }\right)J_{x}\left(h,\beta_{i},r_{i},a_{i}\right)$$

where *ŝ*_*i*_(**x**; *h*, ***β***_*i*_, **r**_*i*_, **a**_*i*_) is used to denote the estimated intensity in the space *s* for the *i* th scan in voxel **x**, where **x**′ is given by Eq. (2), where *f*_*i*_(**x**′) denotes a spline-interpolated value from the regular grid *f*_*i*_ at the (non-integer) voxel index **x**′ and where *J*_**x**_(*h*, ***β***_*i*_, **r**_*i*_, **a**_*i*_) denotes the Jacobian determinant (at voxel **x**) of the mapping in Eq. (2). We will use *ŝ*_*i*_(*f*_*i*_, *h*, ***β***_*i*_, **r**_*i*_, **a**_*i*_) when we refer to the entire collection of (all **x**) voxels.

Equivalently when resampling an image in the space of *s* into that of an observed image *f*_*i*_ it is performed by

(5) $${\hat{f}}_{i}\left(x;s_{i},h,\beta_{i},r_{i},a_{i}\right)=s_{i}\left(x^{\prime }\right)J_{x}^{-1}\left(h,\beta_{i},r_{i},a_{i}\right)$$

and similarly ${\hat{f}}_{i}\left(s_{i},h,\beta_{i},r_{i},a_{i}\right)$ will be used to denote the whole volume *s*_*i*_ resampled into the space of *f*_*i*_.

### Predicting diffusion data

One difficulty with registering diffusion data is to choose a suitable cost-function. The images acquired with different diffusion encoding (both *b*-value and direction) are inherently different. This makes it non-ideal to use straightforward measures of image similarity (see for example Bastin, 2001, Rohde et al., 2004). In addition, all the diffusion weighted images will be affected by distortions so there is no geometrically faithful DWI that can be used as a reference for registration.

In the present paper we suggest instead to make predictions for each diffusion weighted image (characterized by a *b*-value and a direction) and to use that as a target. As long as the prediction is closer to the true (undistorted) space than is the current estimate of the corrected observed image, this should bring the observed image closer to the true space. We suggest using a Gaussian Process (GP) described in Andersson and Sotiropoulos (2015) to make predictions. In brief, it assumes that the signal varies smoothly, both as a function of *b*-value and diffusion gradient direction, and uses linear combinations of the observed data to make predictions. The smoothness is determined from hyperparameters whose values are determined directly from the data. The GP is designed to be axially symmetric, which means that the predictions for diffusion gradients **g** and − **g** would be identical. Correspondingly the weights assigned to data acquired with **g** and − **g** are identical when making predictions about the signal given some other diffusion gradient **g**′. However we would expect **g** and − **g** to cause very different eddy currents. If one assumes that the EC is a linear function of the preceding diffusion gradient they would be the negation of each other.

To get an intuition for how this might work, consider the extreme example where there is no smoothness but where the GP still enforces axial symmetry. In that case the predictions for **g** and − **g** are simply the average of the images observed for **g** and − **g**. That average will be a blurred image in the average space of the two images, and if assuming that the EC is a linear function of the gradients, that average space will be distortion-free space. Registering the observed images to their average will not yield a perfect result due to the blurriness of the average, but it will nudge both images in the right direction. For the next iteration the prediction will be the average of the “nudged” images, *i.e.*, it will still be in distortion-free space but it will now be a less blurred representation of that space. After a few iterations of this, both images will be in minimum-distortion space and the prediction (average) will be sharp.

The above is *only* an attempt to explain why the strategy we suggest might be expected to work. Importantly for any real data, the hyperparameters will be such that the predictions are a smooth function of gradient direction and, in the case of multi-shell data, *b*-value. That smoothness means that one does *not* need to acquire data for both **g** and − **g**, which would from a diffusion signal perspective be redundant and would halve the angular resolution for a given acquisition time (see section Data requirements for eddy for specific data requirements).

### The registration algorithm

We are now ready to describe the algorithm, which can be summarized briefly as

**Figure:**
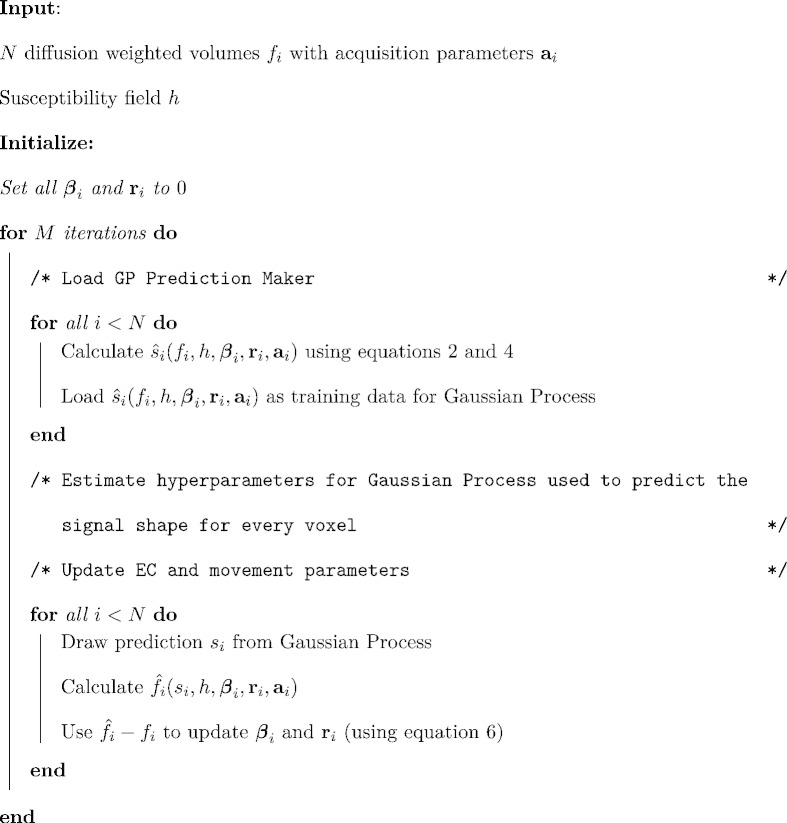

The algorithm is explained graphically in Fig. 1.

**Fig. 2:**
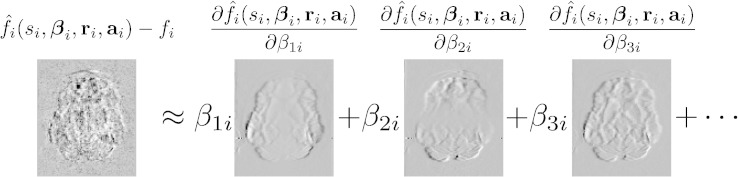
The observed difference between the prediction (${\hat{f}}_{i}\left(s_{i},\beta_{i},r_{i},a_{i}\right)$) and the observation (*f*_*i*_) is modeled as a linear combination of the partial derivatives of the prediction w.r.t. to the parameters (***β***_*i*_) defining the EC-field and the rigid body parameters (**r**_*i*_). For space reasons the figure only shows the first three parameters of ***β***_*i*_. The update is calculated by solving the equation shown in the figure for [*β*_1*i*_*β*_2*i*_*r*_61_] in a least-squares sense after which it is added to the previous estimate of [***β***_*i*_**r**_*i*_].

The final thing that needs explaining is how ${\hat{f}}_{i}-f_{i}$ is used to update the estimates of ***β***_*i*_ and **r**_*i*_. The update is performed by modeling the observed difference ${\hat{f}}_{i}-f_{i}$ as a linear combination of the partial derivatives of ${\hat{f}}_{i}$ with respect to the elements of ***β***_*i*_ and **r**_*i*_.

(6) $$\left[\begin{array}{l} \beta_{i}^{\left(k+1\right)}\\ r_{i}^{\left(k+1\right)} \end{array}\right]=\left[\begin{array}{l} \beta_{i}^{\left(k\right)}\\ r_{i}^{\left(k\right)} \end{array}\right]-{\left(D^{T}D\right)}^{-1}D^{T}\left({\hat{f}}_{i}-f_{i}\right)$$

where ***β***_*i*_^*k*^ and ***β***_*i*_^*k* + 1^ denote the EC parameter estimates for volume *i* after the *k*th and the (*k* + 1)th iteration respectively. **D** is the matrix

(7) $$D=\left[\begin{array}{llllll} \frac{\partial {\hat{f}}_{i}}{\partial \beta_{1i}} & \ldots & \frac{\partial {\hat{f}}_{i}}{\partial \beta_{ni}} & \frac{\partial {\hat{f}}_{i}}{\partial r_{1i}} & \ldots & \frac{\partial {\hat{f}}_{i}}{\partial r_{6i}} \end{array}\right]$$

where $\frac{\partial {\hat{f}}_{i}}{\partial \beta_{ji}}$ is an *m* × 1 column vector with the derivative of ${\hat{f}}_{i}$ with respect to the *j* th eddy current parameter for the *i* th scan for all *m* voxels and correspondingly for $\frac{\partial {\hat{f}}_{i}}{\partial r_{ji}}$ and the *j* th movement parameter. In both Eqs. (6), (7) the dependence of ${\hat{f}}_{i}$ on *s*_*i*_, *h*, ***β***_*i*_, **r**_*i*_ and **a**_*i*_ is implicit. Note also that the derivatives are calculated at the [(***β***_*i*_^(*k*)^)^T^ (**r**_*i*_^(*k*)^)^T^]^T^ point in parameter space. This means that the “cost-function” for the update is the sum-of-squared differences between the prediction in acquisition space (${\hat{f}}_{i}$) and the actual observations (*f*_*i*_), and that the update is a Gauss–Newton step (Press et al., 2007). The update is explained graphically in Fig. 2.

Each update for a given volume, as performed by Eq. (6), is “checked for success” by resampling ${\hat{f}}_{i}$ to make sure that it results in a reduction of the sum-of-squares of $\left({\hat{f}}_{i}-f_{i}\right)$. If it does not the update will be discarded and that volume will retain its old parameters ([(***β***_*i*_)^T^ (**r**_*i*_)^T^]^T^) for the next iteration. There is no check for convergence of the algorithm, instead it is run for a fixed (though user specified) number of iterations. The reason is that our framework does not follow a classical model fitting paradigm and does not fit the same model in every iteration. The model is refined after every iteration and a higher complexity model is fitted. This results in a cost-function that does not necessarily decrease monotonically, even if the parameter estimation improves from one iteration to the other. Our experience is that it does typically converge after five iterations, and all results in the present paper are based on five iterations.

We have seen data sets with very severe subject movement that have required more than five iterations for convergence, in which case ten iterations have been sufficient. The eddy implementation provides summary statistics such as RMS movement related voxel displacements that can be used to flag potential problem data sets where additional iterations may be necessary.

### Data requirements for eddy

The contrast that drives the registration in eddy is the difference in EC (and hence distortions) between data with similar diffusion signal. Let us assume that **g** and **g**′ are two diffusion gradients selected so that within the gradient scheme **g**′ is one of the gradients with the smallest angle to **g**. The corresponding images (*f*_**g**_ and $f_{g^{\prime }}$) would have similar contrasts and $f_{g^{\prime }}$ would have a large weight when predicting *f*_**g**_. However *f*_**g**_ and $f_{g^{\prime }}$ will also have similar distortions so there will be little difference between the prediction and *f*_**g**_, which means that there is little to drive the registration. If the gradient scheme had contained − **g**′ instead of **g**′ it would have had the same information w.r.t. diffusion, but now the distortions would have been more different and hence there would be more information to drive the registration.

This implies that if diffusion is sampled on the whole sphere, rather than on the half sphere, eddy will be better able to correct for distortions (see http://fsl.fmrib.ox.ac.uk/fsl/fslwiki/EDDY for a graphic explanation). Note that this does not mean that one has to sacrifice anything in terms of angular sampling or acquisition time.

Unfortunately many commonly used diffusion schemes (including some supplied by the scanner vendors) are not on the whole sphere which means that not only are they not ideally suited for eddy, but also that there is a non-zero *average* distortion in the diffusion data compared to the *b*_0_ scan/scans. This can be dealt with by using a 2nd level model for the eddy currents as described in section Second level modeling, but the ideal is to acquire data on the whole sphere.

Another source of information about the distortions is data obtained with similar diffusion weighting but different acquisition parameters so that a given off-resonance field causes different distortions in the two volumes. The simplest example would be two volumes obtained with the same diffusion gradient **g** and opposing PE directions. In that case, the image contrast would be identical and the displacements sign reversed (under the reasonable assumption that the diffusion gradient **g** causes the same eddy currents each time). It is then easy to estimate the distortions from such a pair.

However, this source of information can, similar to above, also be used without having to acquire the same gradient twice. Let us make the same assumption as above, that **g** and **g**′ are two diffusion gradients selected so that within the gradient scheme, **g**′ is one of the gradients with the smallest angle to **g**. If one of these are acquired with opposing PE direction compared to the other there will be more valuable information about the eddy currents in the pair.

It is important to notice also that one can “lose” the information by “double” negation. Let us say that **g** and **g**′ are two gradients with a small angle between them and that in order to augment the information for eddy one decides to instead acquire − **g**′. If one then acquires **g** and − **g**′ with opposing PE-directions, the distortions will again become very similar and one is back to square one.

If one also wants to correct for susceptibility distortions, one needs to acquire at least one pair of *b* = 0 images with reversed phase-encode gradients, one of which can be part of the “main” diffusion data set.

### Second level modeling

Our framework naturally lends itself to regularization of the estimates of the EC fields. Even if this is not generally required, there are special cases where it can be useful. A particular case is when the diffusion gradients have been poorly/unevenly distributed on the diffusion sphere *and* one has not collected data with opposing PE-directions. It should be noted that eddy has no inherent knowledge of the “undistorted space” and just registers all volumes towards an average space. If the diffusion gradients are evenly distributed on the whole sphere, that space will be close to “undistorted space”. In contrast, if for example all volumes have been acquired with a positive *z*-component of the diffusion gradients (*i.e.*, if gradients have been optimized on the northern hemisphere), it is easily realized that the average space will be distorted according to a positive *z*-component. One way to deal with that is to model the EC-estimates as a function of the diffusion gradients with zero intercept. There is a detailed description of the second level modeling in Appendix B.

### Final resampling

For all transforms that are part of the estimation of ***β*** and **r** (*i.e.*, those given by Eqs. (2), (3)), the resampling is performed as described in the Resampling the images section. That resampling typically yields good results, but is unable to recover the full resolution in areas where the distorted images have been compressed, causing the signal from several voxels to pile up in fewer voxels. An alternative was suggested in Andersson et al. (2003), where the signal along a column in the PE-direction was reconstructed in a least-squares sense from two images with opposing phase-encode direction. This resampling strategy is available in eddy for data that supports it, *i.e.*, where data has been acquired with opposing phase-encode directions for each diffusion gradient. In that case, data is first corrected for subject movement using the estimated **r** followed by a pair-wise reconstruction of images where the **K**-matrices (see Andersson et al. (2003) for a definition of **K**) are based on the off-resonance resulting from a combination of susceptibility and eddy currents, and where the solution is regularized using the Laplacian.

Either way the resulting output images will only have been resampled once, incorporating all effects of susceptibility, eddy currents and subject movement.

## Material and methods

### Implementation

The algorithm described above was implemented using the C++ programming language and is publicly released as part of the FSL package (see Smith et al., 2004 and http://fsl.fmrib.ox.ac.uk/fsl/fslwiki/). Parts of the algorithms are computationally intensive so that when used on large sets of data (such as the HCP data with 576 volumes each with a matrix size of 144 × 168 × 111 S.N. Sotiropoulos et al., 2013) execution times become prohibitive. In particular calculating the derivatives used in Eq. (6) is costly because each of the columns of the **D** matrix involves inverting a displacement field as implied in Eq. (3) and then resampling the image volume. Therefore the implementation has been parallelized using OpenMP (OpenMP Architecture Review Board, 2008) and CUDA (Farber, 2011). The CUDA implementation, however, has not been publicly released. Execution times are quite reasonable for the CUDA version where for example the 2 mm FMRIB data (A, 128 96 × 96 × 64 volumes, full description in section FMRIB data) runs in 13 min using a quadratic model for the EC-fields on a MacBook Pro with an NVIDIA GeForce 750 M graphics card. This is comparable to the time it takes to acquire such a data set which starts making it feasible for routine clinical use.

### Data

For the validation we have used data from several different scanners and protocols. They are all from the medium to high range of qualities in terms of resolution and number of directions. On the other hand they are in some cases also quite severely affected by distortions, both susceptibility- and eddy current-induced, for reasons that will be explained under the respective data headings. Each data set has been given a capital letter to denote it by in the remainder of the paper.

#### FMRIB data

These are the protocols recommended for tractography on the FMRIB-center 3 T Siemens Verio scanner. All scanning was performed using monopolar Stejskal–Tanner (ST) diffusion weighting, a single shell with a *b*-value of 1500 s/mm^2^, 60 distinct diffusion gradients optimized on a half sphere and then re-distributed on the whole sphere and four *b* = 0 images interspersed. The total of 64 scans were repeated twice with opposing phase-encode directions (A→P and P→A) and using GRAPPA with an in-plane acceleration of 2. No multi-band (across plane acceleration) was used.

##### 2 mm isotropic data (A)

The 2 mm isotropic data was collected as 96 × 96 × 64 volumes with an echo-spacing of 0.68 ms, an echo-time of 86 ms and a repetition time of 8.9 s.

##### 1.5 mm isotropic data (B)

The 1.5 mm isotropic data was collected as 128 × 128 × 86 volumes with an echo-spacing of 0.76 ms, an echo-time of 91 ms and a repetition time of 18.1 s.

It should be noted that although both the 2 mm and the 1.5 mm data were acquired with 60 directions, the gradient tables were different and there were no directions common to the two data sets.

#### Early stage HCP data (C)

As part of the early testing of protocols in the HCP project data were acquired with four shells. These data were acquired on the 3 T Siemens Connectome Skyra system with the high performance SC72 gradient system capable of gradient strengths of 100 mT/m with special gradient amplifiers (Uğurbil et al., 2013). All scanning was performed on the same subject using monopolar Stejskal–Tanner gradients and four different data sets were collected with *b*-values of 1500, 3000, 5000 and 7000 s/mm^2^, 90 × 106 × 66 volumes and an isotropic resolution of 2 mm. Phase-encoding was in the left-right direction, no in-plane acceleration was performed and the echo-spacing was 0.57 ms. For each *b*-value 150 unique directions were acquired and repeated for both PE-directions. A multi-band factor of 3 was used, the echo times were optimized for each *b*-value and were 61, 69, 79 and 90 ms respectively resulting in repetition times of 2, 2.6, 3 and 3.2 s.

#### 3 T HCP data (D)

These data have been described in great detail in S. Sotiropoulos et al. (2013). It was acquired on the same scanner and with the same Stejskal–Tanner gradients as described above. Three shells with *b*-values of 1000, 2000 and 3000 were acquired with 96 unique directions for each shell. The resolution was 1.25 mm isotropic, the imaging matrix was 144 × 168 × 111 pixels. Phase-encoding was in the left-right direction, no in-plane acceleration was performed, the echo-spacing was 0.78 ms and a multi-band factor of three was used. The entire data set was acquired twice with flipped PE-directions. Importantly, for our purposes, *b* = 0 volumes were interleaved with the dwis such that every sixteenth volume had no diffusion weighting. With a repetition time of 5.5 s it means that a *b* = 0 volume was acquired every 88 s.

#### 7 T HCP data (E)

The HCP 7 T data were acquired on the University of Minnesota 7 T Siemens Magnetom scanner. In brief, data was acquired with a 1.05 mm isotropic resolution in 200 × 200 × 132 volumes with an echo spacing of 0.82 ms, an echo time of 71 ms and a repetition time of 7 s (Vu et al., 2015). A multi-band factor of 2 and an in-plane acceleration factor of 3 was used. Diffusion weighting was achieved using a monopolar Stejskal–Tanner scheme and two shells with *b*-values of 1000 and 2000 s/mm^2^. Each shell contained 128 volumes divided into 64 directions optimized on the whole sphere, acquired twice with opposing phase encode directions (anterior-to-posterior and posterior-to-anterior).

### Analysis

For several of the data sets, the diffusion gradients are duplicated for opposing PE-directions. This offers a way to assess the performance of eddy and also to compare it to a commonly used existing method (eddy_correct in FSL) that uses FLIRT (Jenkinson and Smith, 2001), a 12-dof affine transformation and correlation ratio as a cost-function to register the diffusion weighted images to a *b* = 0 image. Two images acquired with the same diffusion gradient will have the same contrast and any difference between them should be due to differences in distortions and/or measurement error (noise). We therefore ran eddy separately on the data with the two different PE-directions and then calculated the sum-of-squared differences for paired diffusion weighted images.

Data acquired with different PE-directions also differ with respect to different susceptibility-induced distortions and if not corrected these would dominate any comparison between the images. We therefore used RGM and pairs of *b* = 0 (where there will be no eddy current-induced distortions) with different PE-directions to estimate the susceptibility-induced off-resonance field and applied that to the images using spline-interpolation and Jacobian modulation (see section Resampling the images). These “susceptibility-only corrected” pairs were the baseline against which the eddy and eddy_correct methods were compared. The eddy_correct method was modified to use spline interpolation and also to be able to incorporate the susceptibility-field from RGM so as to allow for a single resampling into a space corrected for susceptibility, eddy currents and subject movement in the same way that eddy does.

A series of tests was run on the FMRIB (data sets A and B) and the early HCP data (C) to evaluate different settings for the options in eddy. As described above these tests were performed by running eddy separately on the A → P and the P → A (or L → R and R → L in the case of the HCP 3 T data (C)) data and then compared pairwise to assess how well the correction worked. These settings were

**Estimation of GP hyperparameters** There are several different options for determining the hyperparameters for the Gaussian process that model the diffusion signal. These are maximum marginal likelihood (MML), leave-one-out cross validation (CV) and Geissers's surrogate predictive probability (GPP). For each method data was extracted from 1000 random brain voxels and used for the estimation. Note that this random voxel selection potentially introduces a run-to-run variability to the eddy results, but which can be turned off by specifying a seed at the command level.

**Q-space smoothing** The GP can be seen as a smoothing operation in Q-space. We tested different levels of increased smoothing by multiplying the error-variance estimates (hyperparameter of the GP) by values ranging from 1 (no additional smoothing) to 10.

**Spatial smoothing** Data and predictions were smoothed with a Gaussian filter with FWHM ranging from 0 to 5 mm. N.B. that the filtering is applied only during the estimation phase and *not* to the final resampled results.

**EC model** Different models for the EC-induced fields corresponding to first (four parameters), second (ten parameters) and third (20 parameters) order polynomials were tested. See Appendix A for a complete description of the different models.

**Second level modeling** The EC-parameters were fitted to a first or second order polynomial at the end of each iteration.

**Joint modeling of multi-shell data** When having multi-shell data one can either correct each shell independently or one can model (and correct) them all simultaneously. The latter option is potentially better because the Gaussian process is able to use data from one shell when making predictions about another shell (Andersson and Sotiropoulos, 2015). To test that, we corrected the HCP 3 T data (C) for each shell individually and also jointly for all four shells.

#### Estimation of rotation parameters

The rotation parameters from estimated movement is sometimes used to rotate the diffusion gradients prior to subsequent diffusion modeling (Leemans and Jones, 2009). It is therefore of interest to specifically compare the accuracy of the rotation parameters estimated with eddy to those obtained by eddy_correct. In general it is trivial to register two different *b* = 0 images since the contrast is expected to be identical and since there are no differential eddy current-induced distortions. One can therefore view the rotation parameters estimated for the *b* = 0 volumes as “ground truth” parameters available every 88 s. By plotting the rotation parameters obtained for the diffusion weighted images along with the interleaved “ground truth” parameters obtained for the *b* = 0 volumes one can assess the accuracy of the former. Note that eddy aligns all volumes to the first volume while eddy_correct aligns the two phase-encode directions separately (*i.e.*, to the first and the 289th volume). For this purpose that does not matter as the comparison is anyway made to the nearest *b* = 0 volume.

#### How many directions are needed?

Our framework uses all the available data to drive the correction rather than simply registering volume by volume. Therefore, we used the early HCP data to examine how the correction accuracy was affected by the number of diffusion directions. This was done by subsampling the full 150 directions by each time removing 10% of the directions resulting in a sequence of 150, 135, 121 and so forth down to ten directions. Some directions were more affected by ECs than others and some pairs seemed to result in a greater sum-of-squared difference than others. In order to not have the results unduly affected by that, each subsampling level was repeated ten times, each time using a different sample. When removing directions, care had to be taken to ensure that the remaining directions still sampled the whole sphere reasonably well. Failure to do so could have resulted in a very poor sub-set of directions, both from the perspective of sampling the diffusion signal and the eddy currents. We therefore used a sub-sampling strategy where some directions were first removed randomly, followed by removing one direction at a time, each time removing that which minimized the Coulomb forces of the remaining ones (Jones et al., 1999). This was done for the *b* = 1500 and the *b* = 5000 shells, representing “normal” and high *b*-value acquisitions respectively.

#### 7 T HCP data

The 7 T data (E) is quite unique with their close to 1 mm^3^ resolution and the ensuing “poor” SNR at *b* = 2000 s/mm^2^, and also the quite high spatial frequencies found in the susceptibility off-resonance field (a consequence of the high field strength). Because these data are less representative of the data that is commonly acquired and that workers may wish to use eddy for, they are not used for the analyses described above (*i.e.*, the testing of different models, different levels of smoothing etc). This data is included mainly to demonstrate that we have been able to achieve good results even on data as “difficult” as this.

## Results

For all data sets we performed quantitative and qualitative evaluations when using either the quadratic or the cubic EC models. In the supplementary data there are movies that demonstrate the results comparing before and after correction, comparing the linear to the quadratic EC model and comparing eddy to eddy_correct. The supplementary data furthermore contains tables of the quantitative evaluations. The quantitative results are summarized in the following sections.

### Analysis

**Estimation of GP hyperparameters** Correction accuracy does not seem to depend on the method used to estimate the hyperparameters. Based on that result, leave-one-out cross-validation (CV) was used as default for subsequent analyses owing to its conceptual simplicity. The findings are presented in Table C1 in the supplementary data.

**Fig. 3:**
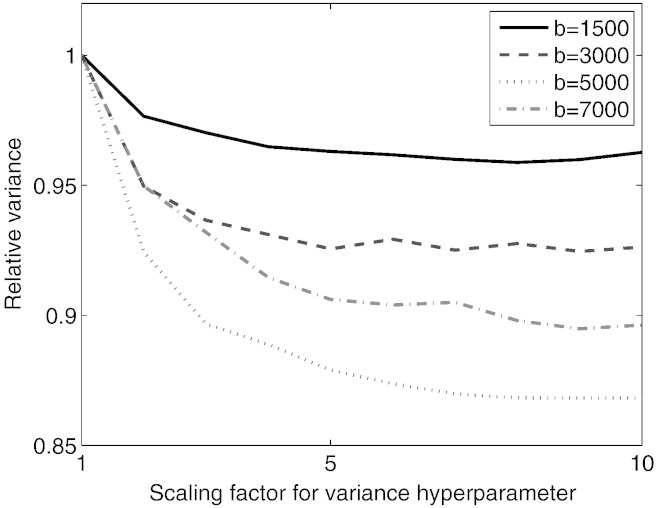
Plot showing how the error variance (as assessed by sum-of-squared differenced of paired images with opposing PE directions) relative to that of no Q-space smoothing depends on the level of Q-space smoothing. The Q-space smoothing is achieved by multiplying the GP-error-variance by a factor greater than 1, and is shown for the range 1 to 10. It can be seen that it has a relatively minor effect on “normal” *b*-values of 1500, but that it has a substantial effect on data acquired with higher *b*-values (5000 and 7000). The plot indicates that by choosing a value of 10 close to optimal results are obtained for all *b*-values. Data set C was used for this figure.

**Fig. 4:**
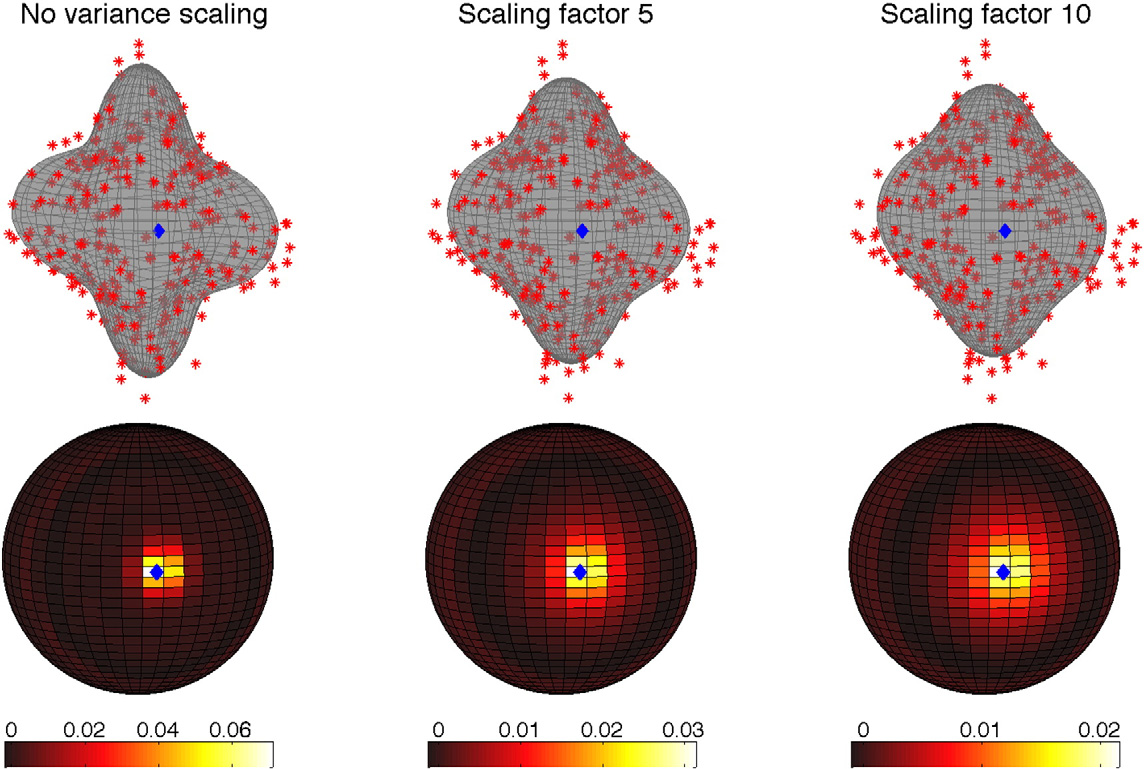
The top row shows the effect of Q-space smoothing, as effected through the error variance hyperparameter of the GP, on the predicted signal. The red markers represent signal observed for different diffusion directions (as distance from the center) and the gray surface represents the predictions made by the GP. The lower row shows the weights given to neighboring points when predicting the signal for the point indicated by the blue marker. The values along the color bars pertain to the (hypothetical) case where there is a “measurement” inside each of the (1681) square patches on the sphere and would scale with the inverse of the number of actual measurements. The relative values when comparing different variance scaling are still valid as are the extent of the kernels on the sphere.

**Q-space smoothing** We noticed a clear dependence of correction accuracy on the level of Q-space smoothing. This is shown in Fig. 3 where it can be seen that especially for higher *b*-values it has a big effect. Based on these findings we decided to use a GP-error-variance scaling factor of 10 in subsequent analyses. Fig. 4 demonstrates the effect this has on the weights used by the GP when making predictions.

**Spatial smoothing** There is some indication for optimal accuracy using a FWHM of 2 mm. Because of the weakness of the effect, we used 0 mm for subsequent analyses. The findings are tabulated in Tables C2, C3 and C4 in the supplementary data.

**Fig. 5:**
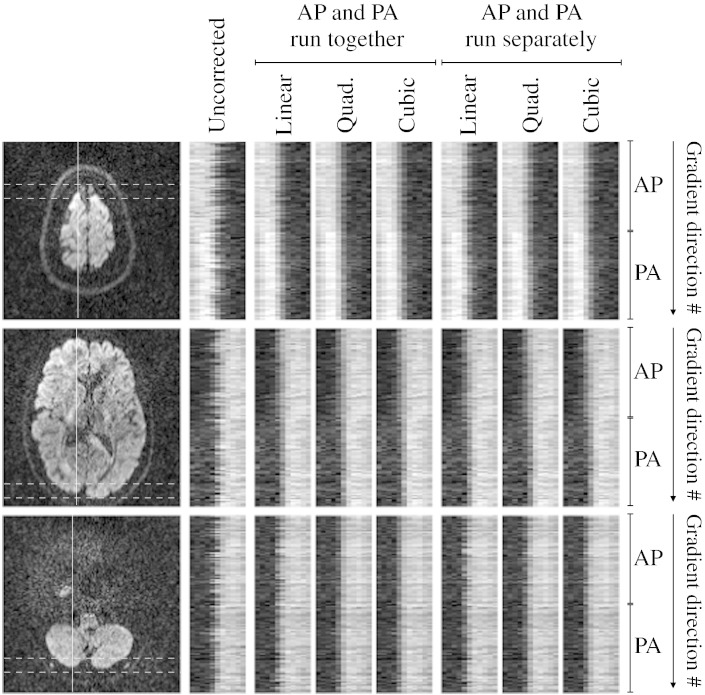
The left column of images show three different slices for the FMRIB 1.5 mm data (B), and for each there is a vertical line that shows where the 1-D profiles were obtained. Perpendicular to that line are two horizontal lines that show the extent of the profiles (how many voxels it was obtained for). The next column shows that profile for each of the 120 diffusion weighted images (divided onto 60 + 60 images with phase-encoding A → P and P → A) without any correction for eddy currents (but corrected for susceptibility using topup). The next three columns show the corrected results when all the data (A → P and P → A) was used to run eddy with, from left to right, linear, quadratic and cubic models for the eddy current-induced field. The final three columns show the results when eddy was run separately for the A → P and the P → A data.

**Fig. 6:**
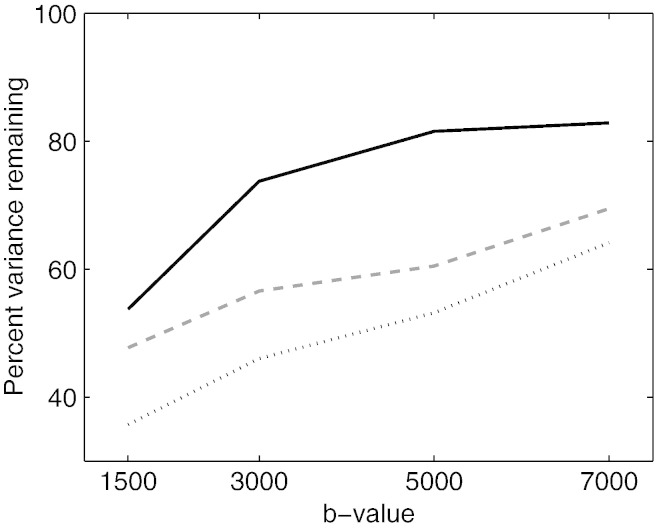
Plots showing error variance relative to only correcting for susceptibility. The curves are *solid black line*: eddy_correct, *dashed gray line*: eddy with linear EC model and *dotted black line*: eddy with quadratic EC model. Data set C was used for this figure.

EC model For all data sets the quadratic model gave substantially better results than the linear one. This can be seen in Fig. 5, Fig. 6 for the FMRIB data (B) and the HCP data (C) respectively. This is further demonstrated in Movie C1, Movie C2 and Tables C2, C3, C4, C5 and C6 in the supplementary data.

**Second level modeling** When using a linear second level model (*i.e.*, when assuming that the EC field has a linear dependence on the diffusion gradient), we obtained worse results than when assuming a quadratic model or when not explicitly assuming any relationship (see Tables C2, C3, C4, C5 and C6 in the supplementary data). For simplicity we decided to not use a second level model as default. The results are tabulated in the supplementary material.

**Joint modeling of multi-shell data** Results are better when jointly modeling multi-shell data compared to when correcting the shells individually. The results are presented in Table C7 in the supplementary material.

#### Comparison to eddy_correct

Examples of comparing the results of eddy to eddy_correct are shown in Fig. 6, Fig. 8 in the main text and in Tables C5 and C6 and Movie C3 in the supplementary material. It is very clear that it is vastly superior on these sets of data, as well as on any other set of data we have tried it on.

#### Estimation of rotation parameters

**Fig. 7:**
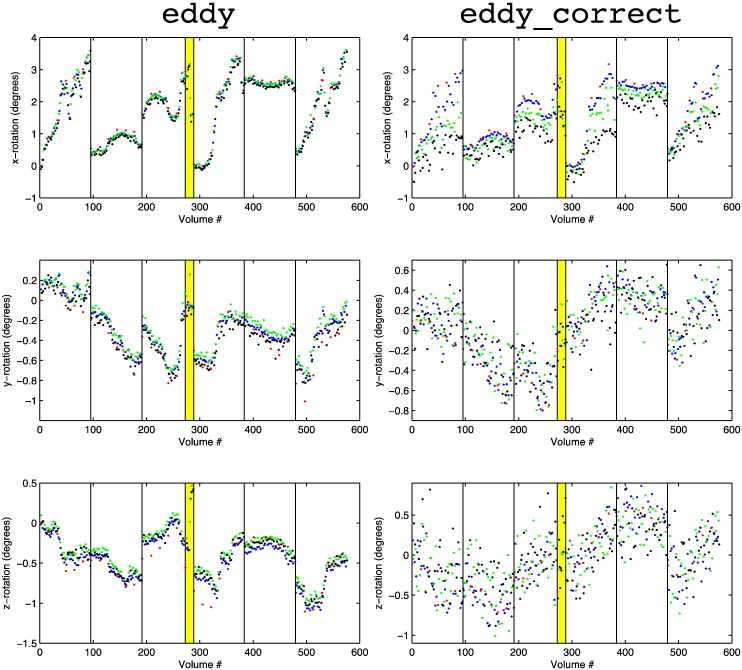
This figure shows the rotation parameters estimated for the *b* = 0 volumes (red) and for the *b* = 1000, *b* = 2000 and *b* = 3000 shells in blue, green and black respectively. The left column shows the estimates from eddy and the right from eddy_correct. The rows, from top to bottom, show rotation around the *x*-, *y*- and *z*-axes respectively. The vertical lines indicate the starts of the six different “sessions” in which the data was acquired. The yellow band indicates a period at the end of the third session during which the subject performed some sudden movements. Note that the ranges of the *y*-axes are not identical for the left and the right column, but that the scale is which allows for a direct visual comparison of the two. Data set D was used for this figure.

In Fig. 7 we show the estimated rotation parameters for the three different shells along with those obtained for the *b* = 0 volumes. It is quite clear that eddy yields rotation parameters with higher accuracy than those of eddy_correct. This is further supported by the correlations between “true” (as assessed by linear interpolation between *b* = 0 rotation estimates) and estimated rotations presented in Table 1.

#### How many directions are needed?

**Fig. 8:**
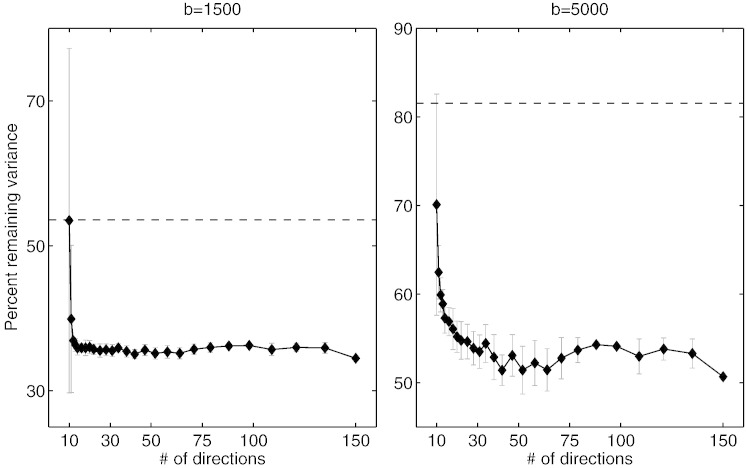
Figure showing the effect of number of directions on the accuracy of the correction. Each point represents ten different subsamples of the size indicated on the *x*-axis (from a total of 150) and the error bars represent the standard deviation across those ten realizations. The accuracy of eddy_correct when applied to the full 150 direction data set is indicated by the dashed line for comparison. It appears that the correction works well down to 15 and 30 directions for the *b* = 1500 and the *b* = 5000 data respectively. Data set C was used for this figure.

Fig. 8 shows how correction accuracy, as measured by sum-of-squared difference by data with opposing PE directions, depends on angular sampling. For a *b*-value of 1500 the corrections remain good and robust (as evidenced by the small standard deviation across the different realizations) down to just 15 directions. For a *b*-value of 5000 results start to get less accurate at 30 directions, though it should be noted that even for as few as ten directions the results are still superior to those obtained by eddy_correct that performs corrections on a volume-by-volume basis.

#### Choice of EC-model

**Fig. 9:**
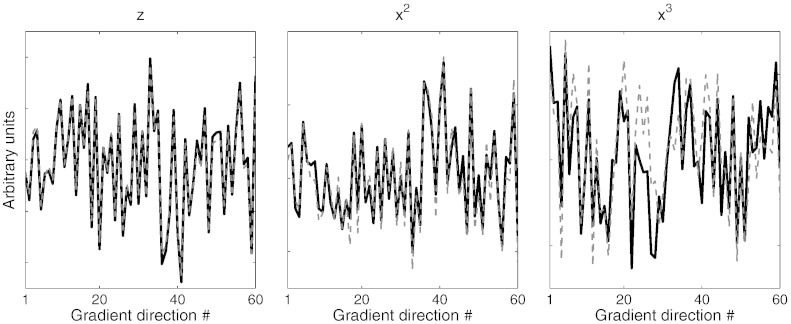
The parameter estimates for the *z*, *x*^2^ and *x*^3^ components of the fields estimated using only the A → P (solid black line) or only the P → A (dashed gray line) FMRIB 1.5 mm data (B). Note that the parameters have been estimated from different data (*i.e.*, no single measurement has been used for both estimates), and yet there is a strong similarity between the estimates with correlations of 0.997, 0.971 and 0.796 respectively for the components seen here.

From the data in Fig. 5, Fig. 6 (and from Tables C5 and C6 and Movie C1, Movie C2 in the supplementary material) there is no doubt that the linear EC model of Jezzard et al. (1998) is insufficient to explain the distortions in the data used for this paper. This is hardly surprising given that modern gradients are not linear in the first place and rely heavily on corrections in the reconstruction process to recover geometric fidelity. It is less clear from our data whether it is best to use a quadratic or a cubic model. The data tabulated in the supplementary material mostly indicate very slightly better results for a cubic model. Furthermore, when comparing EC parameter estimates obtained from separate analyses of A → P and P → A (this can be seen as a form of test-retest experiment), we find a very high agreement (see Fig. 9). Correlations between the A → P and P → A estimates were 0.981, 0.985 and 0.997 for the *x*-, *y*- and *z*-components respectively and 0.971, 0.963, 0.994, 0.988, 0.962 and 0.974 for the *x*^2^-, *y*^2^-, *z*^2^-, *xy*-, *xz*- and *yz*-components respectively. The agreement for the cubic parameters was smaller, but far from zero. The average correlation for the cubic components was 0.64 and the range was − 0.19–0.90 where the negative correlation was for the *y*^2^*z*-component and the highest for the *x*^2^*z*-component (see also Fig. 9). This all implies that the estimated cubic parameters are “meaningful” and are not just a result of overfitting. Nevertheless for these data we consider the improvements very marginal and not enough to motivate the doubling in execution time (roughly proportional to the total number of parameters in **r** and ***β***).

#### Does the 2nd level model tell us something?

It is clear that both the linear and the cubic 2nd level models are very successful in describing the eddy currents, but still not necessarily useful in the context of distortion correction within eddy. We therefore recommend against using that option, at least for data of the type that we have used in the present paper.

It is conceivable that for data with very poor SNR/and or data with very bad distortions it might help the algorithm to find the global optimum and hence make it more robust. Anecdotally we have encountered data of this kind (it later turned out that there was a severe hardware problem with the gradients) that we managed to salvage by using a linear 2nd-level model with eddy.

**Fig. 10:**
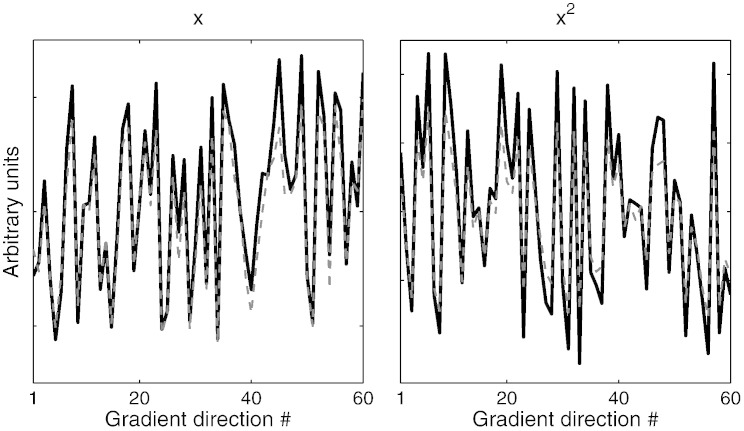
The EC-parameters estimated with a quadratic model for the 1.5 mm (B) was fitted to a second order polynomial of the diffusion gradients (the model given by Eqs. (B3), (B5)). The parameter estimates for the polynomial were used to predict what the EC-parameters should be for the 2 mm data (A). The solid black lines are the estimated EC-parameters for the *x*- (left) and *x*^2^-components (right) estimated directly from the 2 mm data and the dashed gray lines are the predictions made from the 1.5 mm data.

We also tested if we could use the 2nd-level parameters estimated by eddy for one data set to predict the eddy currents for another data set. In Fig. 10 we show the predictions for the 2 mm data (A) based on a second level analysis of the 1.5 mm data (B). It should be noted that the predictions are in no way informed by the 2 mm data and the only thing known to the model is the set of diffusion gradients used for the 2 mm data. Note also that the two data sets were acquired on different days, different subjects, different resolutions, different gradient tables, different PE bandwidths etc and yet the predictions are highly accurate.

#### Q-space smoothing

A result that slightly confused us was that the results improved when applying Q-space smoothing (effected by manipulating the error-variance hyperparameter for the Gaussian Process) when making predictions to drive the registration. We initially assumed that the best results would ensue from using the “optimal” hyperparameters (as assessed by maximizing the marginal likelihood or leave-one-out cross-validation). It does become more clear though when considering the lower row of Fig. 4 which shows the weighting given to any data point on the diffusion sphere. The kernel given by original hyperparameters is very sharp and even for a dataset with 300 points will give almost half the weight to the center-point (*i.e.*, when predicting the signal for a diffusion direction half the information comes from the point itself). Consider taking that to the extreme, which would mean that the prediction and the data were identical, and one realizes that one is now effectively registering each volume to itself which means there is nothing to drive the registration.

## Discussion

### Relationship to earlier work for estimating eddy currents

The previous work that is most similar to that in the current paper is Andersson and Skare (2002) where the sum-of-squared difference between the data and model predictions based on the diffusion tensor (DT) was minimized. There are also important differences

• The DT model is unable to describe data from voxels with complex fiber anatomy acquired with high *b*-values.
• The DT model cannot describe multi-shell data, with *b* > 1500 s/mm^2^ or so.
• In this work we allow for higher order (quadratic and cubic) EC-induced fields.
• Any component of the subject movement that is in the null-space of the log-transformed DT model is invisible to the method in Andersson and Skare (2002) and would hence not be detected/corrected.
• The method in the present paper performs the comparison between prediction and observation in native space of the observation/acquisition.

The last point is relevant for the following reasons

• When using a model *f* to estimate some parameters ***β*** from some data **y** it is typically achieved through min_arg ***β***_∑(*f*_*i*_(***β***) − *y*_*i*_)^2^. *I.e.* one changes the value of ***β***, thereby changing the predictions *f*_*i*_ until they best match the observations **y**. One does *not* change the observations (**y**). However in most image registration algorithms, that is exactly what is done (changing the observations) when transforming an image. This leads to problems since the interpolation will change the summary statistics of the observation error which can bias the parameter estimates (Andersson, 1998). The problem is avoided by our strategy which is much more akin to “proper” parameter estimation.
• Another common artifact in diffusion imaging is signal drop-out caused by macroscopic (subject or pulsatile) movement (Storey et al., 2007). Performing the comparison between the prediction and observation in the native space facilitates the detection of such signal loss (Andersson and Sotiropoulos, 2014) and will be the subject of a future study.

Another method with similarities to ours is that suggested by Zhuang et al. (2013) in which they register the images by comparing them to a subset of the other images acquired with similar direction diffusion gradients. There are however also important differences, in that their definition of “similar direction” is somewhat arbitrary and doesn't adapt to the data at hand (as our GP does), they assume that the EC-induced fields are linear and do not allow for any subject movement.

Other works with similarities to ours are Bodammer et al. (2004) and Shen et al. (2004) that both use the information from pairs of opposing diffusion gradients and Embleton et al. (2010) that instead use pairs with identical diffusion gradients but opposing PE-directions. The similarity is that if opposing diffusion gradients are available eddy will use them. If opposing PE-directions are available eddy will use that too. The difference is that in either case (or if none of the cases above are true), eddy will *also* use the information from all other diffusion weighted images in the set, weighted in such a way that if diffusion gradients are similar (in direction and *b*-value) it will get a larger weight. There is nothing inherently “special” about pairs with opposing PE-directions/diffusion gradients in the way they are used by eddy. The use of all the data makes the process less sensitive to noise, and also to artifacts in one image of the pair. Importantly, this also eliminates the need to “duplicate” acquisitions that would, from a diffusion perspective, yield exactly the same information. This frees up time for a denser sampling of the diffusion sphere or simply shorter scan time. It also does not need to make the assumption (implicit in Bodammer et al., 2004, Shen et al., 2004) that opposing diffusion gradients will give exactly opposing eddy currents. The pair-wise nature of the work by Bodammer et al. (2004), Shen et al. (2004) and Embleton et al. (2010) also makes motion correction more difficult; while the relative positions of the images within a pair can be elucidated there is no clear mechanism for calculating the movement parameters between pairs.

### Avoiding or correcting?

There are several modifications to the Stejskal–Tanner diffusion weighting that aim at reducing/nulling the eddy currents prior to the spatial encoding part of the sequence (for example Alexander et al., 1997, Reese et al., 2003, Finsterbusch, 2009). These modifications yield images with almost no EC-induced distortions straight off the scanner with no need for post-processing and are hence hugely popular and widely used. They have a disadvantage in that they increase the echo-time (in the order of 10 ms) which leads to a loss of signal and SNR. In addition, for non-symmetrical (*i.e.*, non-Stejskal–Tanner) diffusion weighting, the effects of fields concomitant with those intended by the diffusion gradient are not completely re-phased (Baron et al., 2012). This means that the actual diffusion weighting will differ, both in *b*-value and direction, from the intended in a fashion that changes with the distance from the scanner iso-center (*i.e.*, is heterogeneous across the brain). The concomitant-field effect can be minimized for the method by Reese et al. (2003) by tuning the relative sizes of the different gradient lobes, but that is likely to come at an additional cost in terms of echo-time and hence SNR.

Another strategy for reducing the distortions is to use parallel imaging to render the images less sensitive to any off-resonance field (see *e.g.*, Larkman and Nunes (2007) for a review).

By combining EC-nulled diffusion weighting and in-plane parallel imaging one can obtain images with very little, if any, EC-induced distortions. In those cases the linear EC model is likely to be sufficient, and there may even be no need for retrospective distortion correction.

Given their advantages, these methods will certainly continue to be widely used, in particular for clinical applications.

However, these strategies come at some cost in SNR and, in the case of parallel imaging, a trade-off between the level of in-plane parallel imaging and multi-band factor. So, for instance, in the HCP project (Uğurbil et al., 2013, Sotiropoulos et al., 2013b) it was decided against using either twice re-focused diffusion encoding or in-plane acceleration. The use of ST encoding was motivated by a desire for shorter echo-time and better SNR as discussed above. Multi-band was chosen over in-plane acceleration since it meant acquiring higher angular resolution in a given time. We believe that other groups facing similar considerations might make the same choice and thus also be affected by quite severe distortions.

In addition, even when using EC-nulled sequences there is still a possibility of subject movement, in which case the method presented in this paper will be useful.

Another strategy for reducing distortions is reduced field of view (rFOV) imaging (see for example McNab et al., 2013b or Mohammadi et al., 2014). These methods are particularly suited for cases where one wishes to study a specific part of the brain with very high resolution. If one can additionally restrain subject movement (as for example on *ex vivo* samples or sedated subjects McNab et al., 2013b) one can potentially completely avoid any resampling (interpolation) of the data, thereby avoiding the ensuing loss of resolution.

### Measuring or estimating?

There are a multitude of methods that attempt to measure the EC-induced fields, as opposed to try to estimate them from the distorted data. Broadly these can be divided into those that calibrate them “once and for all” (see *e.g.*, Horsfield, 1999, Bastin and Armitage, 2000, Chen et al., 2006, Truong et al., 2011, Wilm et al., 2011, Xu et al., 2013, Chan et al., 2014) and those that use navigators to measure them at every acquisition (Jezzard et al., 1998, Calamante et al., 1999). Of these, Truong et al. (2011), Wilm et al. (2011), Xu et al. (2013) and Chan et al. (2014) are of special interest as they characterize the full time course of the EC-induced fields, and hence offer information that cannot easily be calculated from distorted magnitude images (see also Calamante and Connely, 2012). They are based on characterizing the EC-fields after playing out the desired sequence of diffusion gradients (Truong et al., 2011, Chan et al., 2014) or a polynomial basis for diffusion gradients (Xu et al., 2013) using multiple echo-time fieldmaps (Truong et al., 2011), phase difference between reversed diffusion gradients (Xu et al., 2013) or field probes (Wilm et al., 2011, Chan et al., 2014). One advantage of these “calibration methods” is that since the temporal evolution of the EC-fields is “known” before the scanning, the information about constant and linear (spatially) components can be used to modify the readout accordingly (gradient pre-emphasis) or apply the correction in *k*-space on a per-echo basis, which would potentially also correct for dispersion along the PE direction. It is not clear however how significant that effect is in “real data” and for example in our work on the HCP project we only briefly saw evidence of that in our 7 T data before an upgrade (for unrelated reasons) of the SC72 gradient coils. A slight disadvantage of calibration based methods is the need for calibration scans using specialized sequences (or hardware) and the uncertainty about how often it needs to be repeated.

Even if measuring the eddy currents is preferred, that doesn't address the problem of subject movement, unless one has some strategy for measuring that too (Maclaren et al., 2013). If not, it would be feasible to combine a measured, online-corrected, eddy current sequence with movement estimation and correction using eddy. In this context it is worth noting the results in section Estimation of rotation parameters, which show that eddy estimates movement parameters accurately independent of *b*-value (at least up to *b* = 3000).

It is also possible to feed measured values, or values previously estimated using eddy, as starting values to eddy. The results shown in Fig. 10 demonstrate that to a first approximation the EC-induced distortions are a function only of the diffusion gradients and do not have as strong a dependence on echo time, matrix size *etc.* Hence one can store a file of second level EC parameters and use those as starting estimates for subsequent runs of eddy, potentially making it more robust to for example very high *b*-value data with low SNR.

We suspect that ultimately the choice between using a retrospective method such as eddy or an online method based on calibration data, will be determined by whether the scanner vendors decide to provide the necessary hardware, sequences and reconstruction software for the latter to be a convenient option.

### Final resampling

Any distortions that cause a compression of the whole, or parts, of the FOV leads to a loss of resolution. This is an inevitable consequence of the distortion itself rather than of the interpolation used for the resampling. Especially when using a “high fidelity” interpolation such as for example cubic splines (Unser et al., 1993a, Unser et al., 1993b).

If the data was acquired so that each diffusion direction/*b*-value was acquired twice with opposing phase-encoding this can be partially counteracted as described in section Final resampling. We do not present any results pertaining to that resampling method in the present paper, but we have seen promising results when applied to very high resolution and distortion data from the 7 T scanner (Vu et al., 2015).

### Relevance of this work to “clinical” data

All the data used in the validation in the present paper can be seen as “medium- to high-end” data for research purposes. Hence, the conclusions we draw should not be extrapolated to more typical “clinical” data without further investigation. It is for example not clear if our finding, that a higher order EC-model better models the data, is true also for such data. A typical clinical protocol will have lower resolution and/or in-plane acceleration which means that distortions will be less severe. It may also employ an EC-nulled diffusion weighting that will yield less severe EC-fields and hence also less distortions. If distortions are overall smaller the second order terms will become smaller and may cease to be relevant.

Another limitation is that we have only used Siemens scanners. Different manufacturers may employ different coil-designs, amplifiers, gradient pre-emphasis etc that may all affect the magnitude and nature of the EC-induced fields.

Hence, the findings in the present paper should not be taken to mean that one always needs to correct for higher order EC-induced fields. On the other hand when applying our second-order model to simulated data with only linear EC-fields (see Graham et al. (in press) for details) it correctly estimates a linear field with displacements very close to the simulated true ones. Hence, it seems that there is no “harm” in applying eddy with a higher order EC model even to data with small and purely linear eddy current distortions.

### Current limitations and future work

There are additional effects of subject movement on diffusion data that have not been covered in the present paper. We already mentioned above the signal loss associated with subject/pulsatile movement co-occurring with the diffusion weighting part of the sequence (see for example Pierpaoli, 2011). We are currently working on a method to identify and correct for such dropout (Andersson and Sotiropoulos, 2014) using the framework presented here.

Another extension that is currently being planned is slice-to-volume realignment for the cases where rapid head movement means that the acquired slices do not match when simply stacked up to a volume (Kim et al., 1999).

There is currently no model within eddy that takes spin-history effects into account.

The current implementation of the Gaussian Process can only model data that resides on one or more spheres, *i.e.*, it cannot be used for example on Cartesian DSI data. This is another topic of future investigation.

Finally, given the severe nature of the distortions in for example the WU-UMinn HCP data, the interaction of subject movement and the susceptibility-induced off-resonance field has again become an issue. When a subject moves inside the scanner the resulting off-resonance field will to a first approximation follow the subject. But when there is rotation around an axis non-parallel to the magnetic flux, that approximation does not quite hold and the field changes even in a subject-framework. We have previously modeled this for the fMRI (GE-EPI) case (Andersson et al., 2001) and we plan to adapt that method to diffusion within the eddy framework.

### Conclusion

We have developed a method for estimation of EC-induced fields and subject movement from diffusion images and for correction of these effects along with effects of a susceptibility-induced off-resonance field. Our experience is that it has worked very well for any data we have encountered so far, including *b*-values up to 10,000 and pixel sizes down to 1.05 × 1.05 × 1.05 mm^3^. It is presently being used to correct for EC-distortions and movement in the WU-UMinn HCP (Van Essen et al., 2013, Sotiropoulos et al., 2013b, Vu et al., 2015), the MGH HCP (Setsompop et al., 2013), the Whitehall (Filippini et al., 2014) and the UK Biobank (http://imaging.ukbiobank.ac.uk) studies.

The following are the supplementary data related to this article.

Movie C1 N.B. This is a still from the attached movie movie_2mm_FMRIB.gif. The movie shows three slices through the 2 mm FMRIB data. The bottom row shows data corrected only for susceptibility, the middle row after correction with eddy assuming a linear EC model and the top row assuming a quadratic model. Data set A was used for this movie.

Movie C2 N.B. This is a still from the attached movie movie_15mm_FMRIB.gif. The movie shows three slices through the 1.5 mm FMRIB data. The bottom row shows data corrected only for susceptibility, the middle row after correction with eddy assuming a linear EC model and the top row assuming a quadratic model. Data set B was used for this movie.

Movie C3 N.B. This is a still from the attached movie movie_old_HCP.gif. The columns show data acquired with, from left to right, *b*-values of 1500, 3000, 5000 and 7000. The bottom row shows data corrected only for susceptibility, the middle row shows data additionally corrected using eddy_correct and the top row after correction with eddy. Data set C was used for this movie.

Movie C4 N.B. This is a still from the attached movie movie_7T.gif. The left hand side of the movie shows the HCP 7T data acquired with a *b*-value of 1000 and the right hand side with a *b*-value of 2000. The bottom row shows data corrected only for susceptibility and the top row after correction with eddy. Data set E was used for this movie.

Supplementary material.

## Figures and Tables

**Table 1:** This table shows the correlation between “true” (see text) and estimated rotation parameters for eddy and eddy_correct. Note how eddy performs better than eddy_correct for every rotation axis and every *b*-value. Note also the clear degradation of the performance for eddy_correct with increasing *b*-value. This does not appear to be the case for eddy and *b*-values up to 3000.

Method:	Eddy_correct			Eddy		
*b*-value	x-rot	y-rot	z-rot	x-rot	y-rot	z-rot
1000	0.952	0.930	0.866	0.960	0.947	0.919
2000	0.885	0.865	0.740	0.964	0.942	0.922
3000	0.750	0.767	0.626	0.960	0.937	0.916

## Appendix A Terms and definitions

In this appendix we will explain in greater detail some of the terms and definitions from Definition of terms section.

*s*: *Reference/model space* is used to denote a space given by a FOV and an orientation in which the images are free of any distortions or effects of movement. It is also used to denote any prediction made by the Gaussian process in this space, so for example *s*_*i*_ would denote a prediction for the *i*th diffusion weighted image in reference space.
*f*: *Observed/Acquired image* is used to denote the observed image, and the space associated with it, so that for example *f*_*i*_ denotes the *i*th acquisition/volume in the data set. Note that each observation is associated with a unique space given by the eddy currents and subject movement for that particular volume. This is in contrast to *s* which denotes a single (undistorted) space and the only difference between *s*_*i*_ and *s*_*j*_ is the contrast.
a: *Acquisition parameters* the acquisition parameters that are relevant for eddy are the direction of the phase encoding and the total readout time (here defined as the time between the acquisition of the center of the first and last echoes). Internally **a** is divided into **a** = [**p** *t*] where **p** is a unity length 1 × 3 vector defining the PE direction (such that for example [1 0 0], [− 1 0 0], [0 1 0] and [0 − 1 0] denote R → L, L → R, P → A and A → P PE direction respectively) and where *t* denotes the readout time (in seconds). Note that **p** can also be oblique such as for example in PROPELLER acquisitions (Skare et al., 2006). Different scans in a set of diffusion images may have different acquisition parameters so **a**_*i*_ denotes the acquisition parameters for the *i*th scan.
**r**: *Rigid body transformation* different workers/software differ in how they define rigid body parameters and the ensuing transform. One of the outputs of eddy is a text-file with movement parameters for each image and in order for others to interpret and use these correctly (for example for rotation of diffusion gradients) we need to define clearly how these are used. The rigid body parameters are denoted by **r**, an 1 × 6 vector with three translations followed by three rotations. Let us assume that a given **r** maps an image/space S onto an image/space Q. Let us further assume two matrices $M_{S}$ and $M_{Q}$ that map a voxel-coordinate vector into a mm-coordinate vector for S and Q respectively and that these map the geometric center of respective space onto the mm-coordinate [0 0 0]^T^. Then the mapping is performed by transforming a voxel-coordinate **x** in the space of Q to a voxel coordinate **x**′ in S according to
  (A1) $$\left[\begin{array}{l} x^{\prime }\\ 1 \end{array}\right]=M_{S}^{-1}{\left[\begin{array}{ll} R & t\\ 0^{T} & 1 \end{array}\right]}^{-1}M_{Q}\left[\begin{array}{l} x\\ 1 \end{array}\right]$$
  where **0**^T^ is a 1 × 3 vector with zeros, **t** is a vector with the first three elements of **r**, where **R** = **R**_*x*_**R**_*y*_**R**_*z*_ and where
  (A2) $$R_{x}=\left[\begin{array}{ccc} 1 & 0 & 0\\ 0 & \cos\theta & \sin\theta \\ 0 & -\sin\theta & \cos\theta \end{array}\right]$$
  where *θ* (in radians) is the fourth element of **r** and correspondingly for **R**_*y*_ and **R**_*z*_ with *θ* the fifth and sixth elements respectively. Each scan is associated with a unique set of movement parameters so **r**_*i*_ denotes the estimate of the movement parameters for the *i*th scan.
***β*** and *e*(***β***): *Eddy current-induced field* ***β*** is a set of parameters that uniquely define an EC-induced field *e*(***β***). The EC-induced field model can be linear, quadratic or cubic corresponding to ***β***s with four, ten and twenty elements respectively. For the linear case the three first (of four) elements of ***β*** specify linear gradients (in Hz/mm) in the *x*-, *y*- and *z*-directions respectively and where the zero-crossing of each gradient coincides with the center of the FOV. The fourth element is a field-offset (in Hz) which models any deviation between the center of the FOV and the iso-center of the scanner. For the other EC models the structure of ***β*** is similar with ***β*** = [*x y z x*^2^ *y*^2^ *z*^2^ *xy xz yz* offs] for the quadratic and ***β*** = [*x y z x*^2^ *y*^2^ *z*^2^ *xy xz yz x*^3^ *y*^3^ *z*^3^ *x*^2^*y x*^2^*z xy*^2^ *y*^2^*z xz*^2^ *yz*^2^ *xyz* offs] for the cubic model. Each scan is associated with a unique set of EC parameters so ***β***_*i*_ denotes the EC parameters for the *i*th scan.
*h*: *Susceptibility induced off resonance field (Hz)* this is not calculated by eddy, but will be used if supplied as an input. It is assumed to be in the same space as the first *b* = 0 image supplied to eddy, which will be automatically fulfilled if it was estimated by topup and that same *b* = 0 image was the first of those supplied to topup. Hence, it can be said to help define the reference/undistorted space as the first *b* = 0 image after distortion correction by *h*.
*ω*(*ψ*, **r**): *Rigid body transformed off-resonance field (Hz)* this is the off-resonance field *ψ* after rigid body transformation with the parameters **p**.
**d**(*ψ*, **a**): *Voxel displacement field* given a field (*ψ*) in Hz and the acquisition parameters (**a** = [**p** *t*]) it is trivial to calculate a voxel displacement field. If *ψ*_**x**_ denotes the off-resonance field at voxel **x** = [*x y z*] then the voxel-displacement at **x** is
  (A3) $$d_{x}={\left[\begin{array}{lll} tp_{1}\psi_{x} & tp_{2}\psi_{x} & tp_{3}\psi_{x} \end{array}\right]}^{T}$$
  and **d**(*ψ*, **a**) is simply the collection of **d**_**x**_s for all voxels **x**.

## Appendix B Second level model

The result of each iteration of the algorithm described in The registration algorithm section and Fig. 1 is an updated set of parameter vectors ***β***_*i*_ where *i* indexes the volumes in the diffusion data set. Each of the elements in these vectors pertains to a particular “basis field”. If one for example considers the first element in ***β***_*i*_ (which will be denoted by *β*_*i*_^(1)^ in this appendix. Please note that this is different to in The registration algorithm section where we used the ^(*j*)^ notation to denote iteration # *j*.) it specifies how strong the linear *x*-component is in the EC-induced off-resonance field for volume *i*. A new vector can be created by stacking the first element of each ***β***_*i*_, *i.e.*,

(B1) $$\beta^{\left(1\right)}=\left[\begin{array}{c} \beta_{1}^{\left(1\right)}\\ \beta_{2}^{\left(1\right)}\\ \vdots \\ \beta_{N}^{\left(1\right)} \end{array}\right]$$

which is now a vector that contains the strength of the linear *x*-component of the EC-fields for each of the *N* diffusion weighted volumes.

When a second level model is used each such vector of first level estimates are modeled as a function of the diffusion gradients, *i.e.*,

(B2) $$\beta_{i}^{\left(j\right)}=f\left(g_{i};\theta^{\left(j\right)}\right)$$

where *β*_*i*_^(*j*)^ denotes the first level estimate of the *j*th parameter from the *i*th volume, **g**_*i*_ denotes the diffusion gradient (strength and direction) for the *i*th volume and ***θ***^(*j*)^ indicates the vector of second level parameters for the *j*th first level parameter. In the current eddy implementation there are two options for *f*, both given by

(B3) $$\left[\begin{array}{c} \beta_{1}^{\left(j\right)}\\ \beta_{2}^{\left(j\right)}\\ \vdots \\ \beta_{N}^{\left(j\right)} \end{array}\right]=X\theta^{\left(j\right)}+\epsilon$$

where when assuming a linear relationship between diffusion gradients and eddy currents **X** is given by

(B4) $$X=\left[\begin{array}{ccc} g_{1}^{\left(x\right)} & g_{1}^{\left(y\right)} & g_{1}^{\left(z\right)}\\ g_{2}^{\left(x\right)} & g_{2}^{\left(y\right)} & g_{2}^{\left(z\right)}\\ \vdots & \vdots & \vdots \\ g_{N}^{\left(x\right)} & g_{N}^{\left(y\right)} & g_{N}^{\left(z\right)} \end{array}\right]$$

and when assuming a quadratic relationship by

(B5) $$X=\left[\begin{array}{ccccccccc} g_{1}^{\left(x\right)} & g_{1}^{\left(y\right)} & g_{1}^{\left(z\right)} & {\left(g_{1}^{\left(x\right)}\right)}^{2} & {\left(g_{1}^{\left(y\right)}\right)}^{2} & {\left(g_{1}^{\left(z\right)}\right)}^{2} & g_{1}^{\left(x\right)}g_{1}^{\left(y\right)} & g_{1}^{\left(x\right)}g_{1}^{\left(z\right)} & g_{1}^{\left(y\right)}g_{1}^{\left(z\right)}\\ g_{2}^{\left(x\right)} & g_{2}^{\left(y\right)} & g_{2}^{\left(z\right)} & {\left(g_{2}^{\left(x\right)}\right)}^{2} & {\left(g_{2}^{\left(y\right)}\right)}^{2} & {\left(g_{2}^{\left(z\right)}\right)}^{2} & g_{2}^{\left(x\right)}g_{2}^{\left(y\right)} & g_{2}^{\left(x\right)}g_{2}^{\left(z\right)} & g_{2}^{\left(y\right)}g_{2}^{\left(z\right)}\\ \vdots & & & & \ldots & & & & \vdots \\ g_{N}^{\left(x\right)} & g_{N}^{\left(y\right)} & g_{N}^{\left(z\right)} & {\left(g_{N}^{\left(x\right)}\right)}^{2} & {\left(g_{N}^{\left(y\right)}\right)}^{2} & {\left(g_{N}^{\left(z\right)}\right)}^{2} & g_{N}^{\left(x\right)}g_{N}^{\left(y\right)} & g_{N}^{\left(x\right)}g_{N}^{\left(z\right)} & g_{N}^{\left(y\right)}g_{N}^{\left(z\right)} \end{array}\right]$$

where for example *g*_*i*_^(*x*)^ denotes the *x*-component of the diffusion gradient associated with the *i*th volume. The error vector **ϵ** is assumed to be drawn from an *iid* Normal distribution.

For either model the first level estimates (***β***^(*j*)^) are fitted to the model given by Eq. (B2), after which the first level estimates are replaced by $\hat{\beta^{\left(j\right)}}$ given by

(B6) $$\hat{\beta^{\left(j\right)}}=X{\left(X^{T}X\right)}^{-1}X^{T}\beta^{\left(j\right)}$$

This replacement is performed after each full iteration.
