# Supplementary figures and images for: An integrated approach to correction for off-resonance effects and subject movement in diffusion MR imaging
Source: Neuroimage. 2016 Jan 15;125:1063–78. doi: 10.1016/j.neuroimage.2015.10.019 (PMC4692656; doi:10.1016/j.neuroimage.2015.10.019)

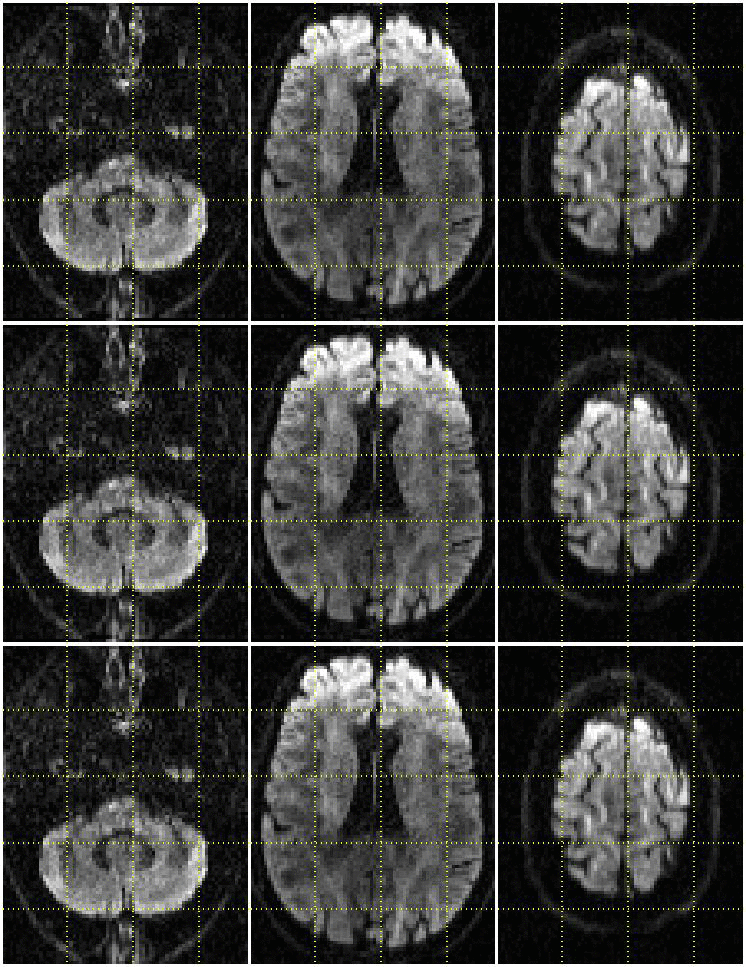

Supplement: Movie C1 — N.B. This is a still from the attached movie movie_2mm_FMRIB.gif. The movie shows three slices through the 2 mm FMRIB data. The bottom row shows data corrected only for susceptibility, the middle row after correction with eddy assuming a linear EC model and the top row assuming a quadratic model. Data set A was used for this movie. [file mmc1.gif]
