# Supplementary material for: An integrated approach to correction for off-resonance effects and subject movement in diffusion MR imaging
Source: Neuroimage. 2016 Jan 15;125:1063–78. doi: 10.1016/j.neuroimage.2015.10.019 (PMC4692656; doi:10.1016/j.neuroimage.2015.10.019)
Supplement: Supplementary file 5 — Supplementary material. [file mmc5.pdf]

C Supplementary material

Here we have put results that we have either deemed superfluous to the main article or which cannot be displayed in print (*i.e.* movies).

C.1 Tables

For all these tables the presented metric is the sum-of-squared pairwise (for pairs with the same diffusion weighting by different PE-direction) differences averaged across all intracerebral voxels (as assessed by mask passed to eddy) and across all pairs.

For all tables we have attempted to help the reader by highlighting the entry with the lowest sum-of-squared difference in the relevant row (or column, depending on the organization of the table).

C.1.1 Dependence on the method for estimating GP hyperparameters

There is no clear preference for either method based on the data in table C1. As we outline in the main text our final preference EC and 2nd level model is to use a quadratic EC model and to not use a 2nd level model. When looking at the results in table C1 for that case (the first three columns) one sees that Cross validation performs best for two of the b-values (5000 and 7000) and second best for b=1500. Based on that and on the appealing conceptual simplicity of leave-one-out cross validation we decided to use that as default and for the remainder of the paper.

C.1.2 Assessing the effect of spatial smoothing

From tables C2, C3 and C4 we conclude that the dependence of the pre-smoothing FWHM is quite weak. For one set of data (the HCP b=1500, table C4) there seems to be a stronger

EC-model:	Quadratic					
2nd level model:	None			Quadratic		
b-value	MML	CV	GPP	MML	CV	GPP
1500	4936	4875	**4873**	5180	5203	5220
3000	**2729**	2752	2742	2829	2824	2864
5000	1850	**1826**	1829	1852	1837	1838
7000	1589	**1588**	1589	1594	1590	1593
EC-model:	Cubic					
2nd level model:	None			Quadratic		
b-value	MML	CV	GPP	MML	CV	GPP
1500	**4926**	5064	4946	5075	5220	5252
3000	**2690**	2720	2708	2745	2826	2827
5000	1824	1817	**1816**	1844	1832	1836
7000	1554	**1553**	1569	1565	1570	1572

Table C1: This table shows the average squared-difference for the HCP 3T data ($\boldsymbol{\mathcal{C}}$) between images obtained with the same diffusion gradient, but different PE-directions. `eddy` has been run with either a second or third order model for the EC fields, with estimated parameters either not regularised at all or regularised using a second order polynomial. These cases were chosen from the greater set of EC models and regularisations as these cases were shown in table C6 to give the best results. For these cases the method for estimating the hyperparameters was varied between Marginal Maximum Likelihood (MML), Cross Validation (CV) and Geisser's Surrogate Predictive Probability (GPP).

EC-model:	linear			quadratic			cubic		
2nd-level model:	none	lin	quad	none	lin	quad	none	lin	quad
FWHM (mm)									
0	511	**513**	**511**	417	**421**	**418**	**411**	**417**	415
1	511	**513**	**511**	417	**421**	**418**	412	**417**	**414**
2	**510**	**513**	**511**	**415**	**421**	**418**	**411**	**417**	**414**
3	**510**	514	512	**415**	422	419	412	418	415
5	515	517	516	418	427	424	415	421	419

Table C2: This table shows the sum-of-squared pairwise differences for the FMRIB 2mm data ($\boldsymbol{\mathcal{A}}$) as a function of the FWHM of a pre-conditioning smoothing applied to the data. It can be seen that for all EC and 2nd level models the dependence on FWHM is very weak and that an "optimum" FWHM is at 2mm.

EC-model:	linear			quadratic			cubic		
2nd-level model:	none	lin	quad	none	lin	quad	none	lin	quad
FWHM (mm)									
0	780	780	780	727	727	725	724	721	720
1	780	780	779	726	726	725	724	721	721
2	770	769	768	716	**716**	**715**	714	**714**	**713**
3	**768**	**767**	**767**	**713**	718	716	**712**	715	**713**
5	770	770	771	720	735	728	721	729	723

Table C3: This table shows the sum-of-squared pairwise differences for the FMRIB 1.5mm data ($\boldsymbol{\mathcal{B}}$) as a function of the FWHM of a pre-conditioning smoothing applied to the data. It can be seen that for all EC and 2nd level models the dependence on FWHM is very weak and that an "optimum" FWHM is at 3mm.

EC-model:	quadratic							
2nd level model:	none				quadratic			
b-value:	1500	3000	5000	7000	1500	3000	5000	7000
FWHM (mm)								
0	5062	2733	1846	1592	**5040**	2820	1851	1593
1	**4921**	2746	1846	1590	**5040**	**2794**	1851	1592
2	5028	**2722**	1825	1576	5212	2853	1828	1576
3	5232	2744	**1806**	**1559**	5596	2866	1819	1556
5	6930	2767	1829	**1559**	5362	2946	**1811**	**1552**

EC-model:	cubic							
2nd level model:	none				quadratic			
b-value:	1500	3000	5000	7000	1500	3000	5000	7000
FWHM (mm)								
0	**4995**	**2702**	1826	**1553**	5239	2774	1840	1568
1	5000	2703	1818	1554	**5128**	2790	1836	1564
2	5145	2710	**1812**	1559	5389	2771	1829	1562
3	5166	2807	1828	1559	5160	2819	**1814**	1552
5	5700	2850	1849	1565	5328	**2770**	1934	**1542**

Table C4: This table shows the sum-of-squared pairwise differences for the HCP 3T data ($\boldsymbol{\mathcal{C}}$) as a function of the FWHM of a pre-conditioning smoothing applied to the data. It can be seen that for the b=1500 data there is some appreciable dependence on FWHM and that the optimum is 0-2mm. It can further be seen that as one goes to higher b-values the optimum FWHM increases with optimums of 1-2mm for 3000, 2-5mm for 5000 and 3-5mm for 7000. However, it can also be seen that the dependence is quite weak for these b-values.

Voxel size (mm)	susc only	`eddy_correct`	eddy								
			linear			quadratic			cubic		
			none	lin	quad	none	lin	quad	none	lin	quad
1.5	1031	995	780	780	780	727	727	725	724	721	**720**
2	845	881	511	513	511	417	421	418	**411**	417	415

Table C5: This table shows the sum-of-squared pairwise differences for the FMRIB data after correction for susceptibility only, using `eddy_correct` and `eddy`. The `eddy` results are tabulated for linear, quadratic and cubic models for the EC-induced fields. For each of those fields the parameters are modeled using different assumptions for how diffusion gradients translate into EC (none, linear or quadratic). We can see `eddy` performs substantially better than `eddy_correct` even when using a linear model (*i.e.* a subset of the model used by `eddy_correct`. It is also clear that the quadratic model for the EC fields perform better than the linear, whereas it is less clear that a cubic model offers any additional advantage. Finally it is not clear that the 2nd level model improves anything. Data sets $\boldsymbol{\mathcal{A}}$ and $\boldsymbol{\mathcal{B}}$ were used for this table.

dependence, and for that there was a preference towards smaller FWHM. Based on this, and for simplicity, we suggest using an FWHM of 0 as default, and that is what has been used for the reminder of the paper.

C.1.3 Assessing the impact of EC-model and 2nd level model

Our conclusion from tables C5 and C6 is that allowing for quadratic EC-fields results in considerably better results. It is not clear that going to cubic EC-fields improve things much further, nor is it clear that employing a 2nd level model helps, and in particular a linear 2nd level model appears to give worse results than when not using a model or when using a quadratic 2nd level model.

	susc	`eddy_`	`eddy`								
	only	`_correct`	linear			quadratic			cubic		
b-value			none	lin	quad	none	lin	quad	none	lin	quad
1500	12970	6969	6188	6248	6218	4630	4481	4501	**4449**	**4449**	4497
3000	5900	4351	3338	3356	3346	2716	2642	2648	2632	**2630**	2641
5000	3436	2802	2078	2095	2086	1827	1842	1838	**1826**	1838	1837
7000	2462	2040	1710	1720	1694	1579	1625	1603	**1575**	1600	1584

Table C6: This table shows the sum-of-squared pairwise differences for the HCP 3T data ($\boldsymbol{\mathcal{C}}$). The second and third columns correspond to susceptibility correction only and correction with `eddy_correct` respectively. The remaining columns correspond to correction with `eddy` using different EC models (first, second or third order polynomials) and models for how diffusion gradients translate into EC (none, linear or quadratic). We can see `eddy` performs substantially better than `eddy_correct` even when using a linear model (*i.e.* a subset of the model used by `eddy_correct`. It is also clear that the quadratic model for the EC fields perform better than the linear, whereas it is less clear that a cubic model offers any additional advantage. Finally it is not clear that the 2nd level model improves anything. The data in the table is also summarized in figure 6.

EC-model:	quadratic				cubic			
b-value:	1500	3000	5000	7000	1500	3000	5000	7000
Modeling option								
Separate	5062	2733	**1846**	1592	4995	2702	**1826**	1553
Joint	**4473**	**2400**	1855	**1438**	**4495**	**2393**	1854	**1433**

Table C7: This table shows the sum-of-squared pairwise differences for the HCP 3T data ($\boldsymbol{\mathcal{C}}$). The first row shows the results when registering each shell separately, and the second row when modeling and registering all four shells simultaneously.

C.1.4 Joint modeling of multi-shell data

The results in table C7 indicate that there is indeed an advantage to modeling the shells together when correcting multi-shell data. Interestingly there seem to be a clear advantage for all shell except for the b=5000 shell for which the correction seems to be similar/slightly worse when modeled in conjunction with the other shells.

Base on these results we recommend modeling all shells together when working with multi-shell data.

C.2 Movies

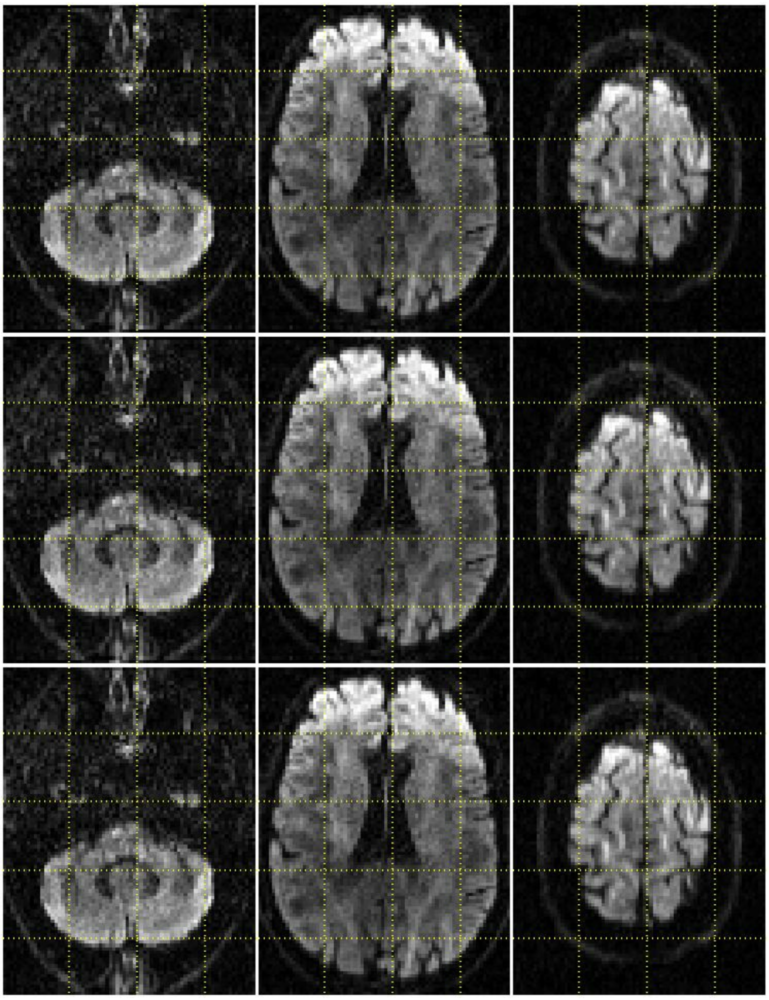

Figure C1: N.B. This is a still from the attached movie movie_2mm_FMRIB.gif. The movie shows three slices through the 2mm FMRIB data. The bottom row shows data corrected only for susceptibility, the middle row after correction with `eddy` assuming a linear EC model and the top row assuming a quadratic model. Data set $\boldsymbol{\mathcal{A}}$ was used for this movie.

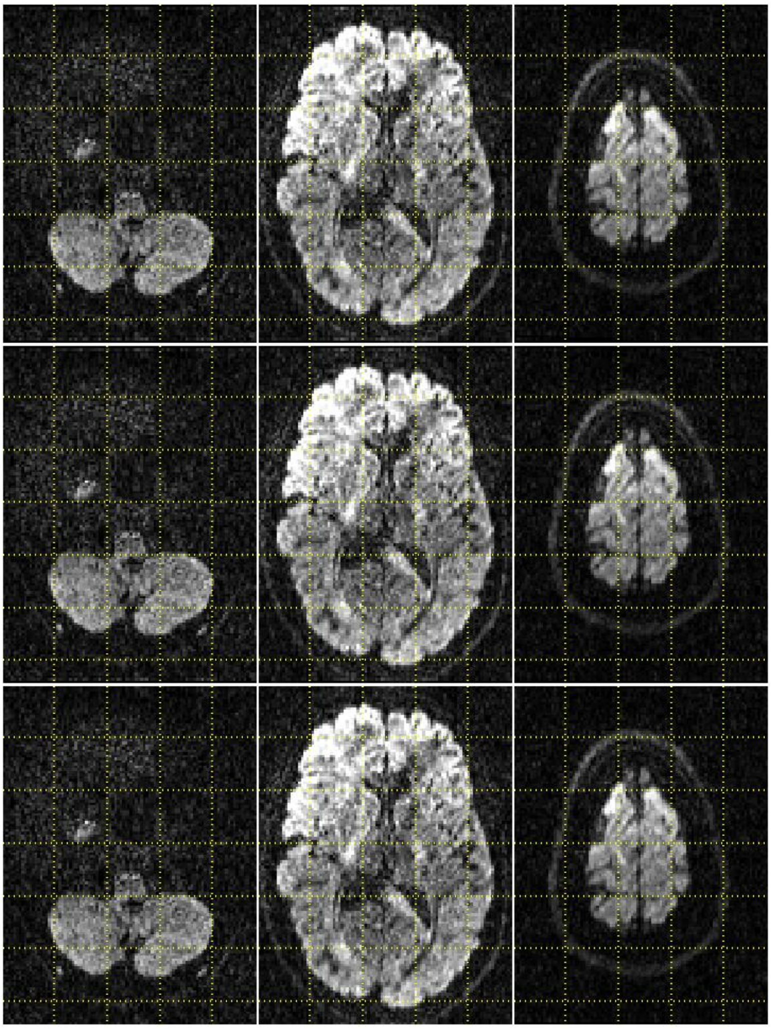

Figure C2: N.B. This is a still from the attached movie movie_15mm_FMRIB.gif. The movie shows three slices through the 1.5mm FMRIB data. The bottom row shows data corrected only for susceptibility, the middle row after correction with `eddy` assuming a linear EC model and the top row assuming a quadratic model. Data set $\boldsymbol{\mathcal{B}}$ was used for this movie.

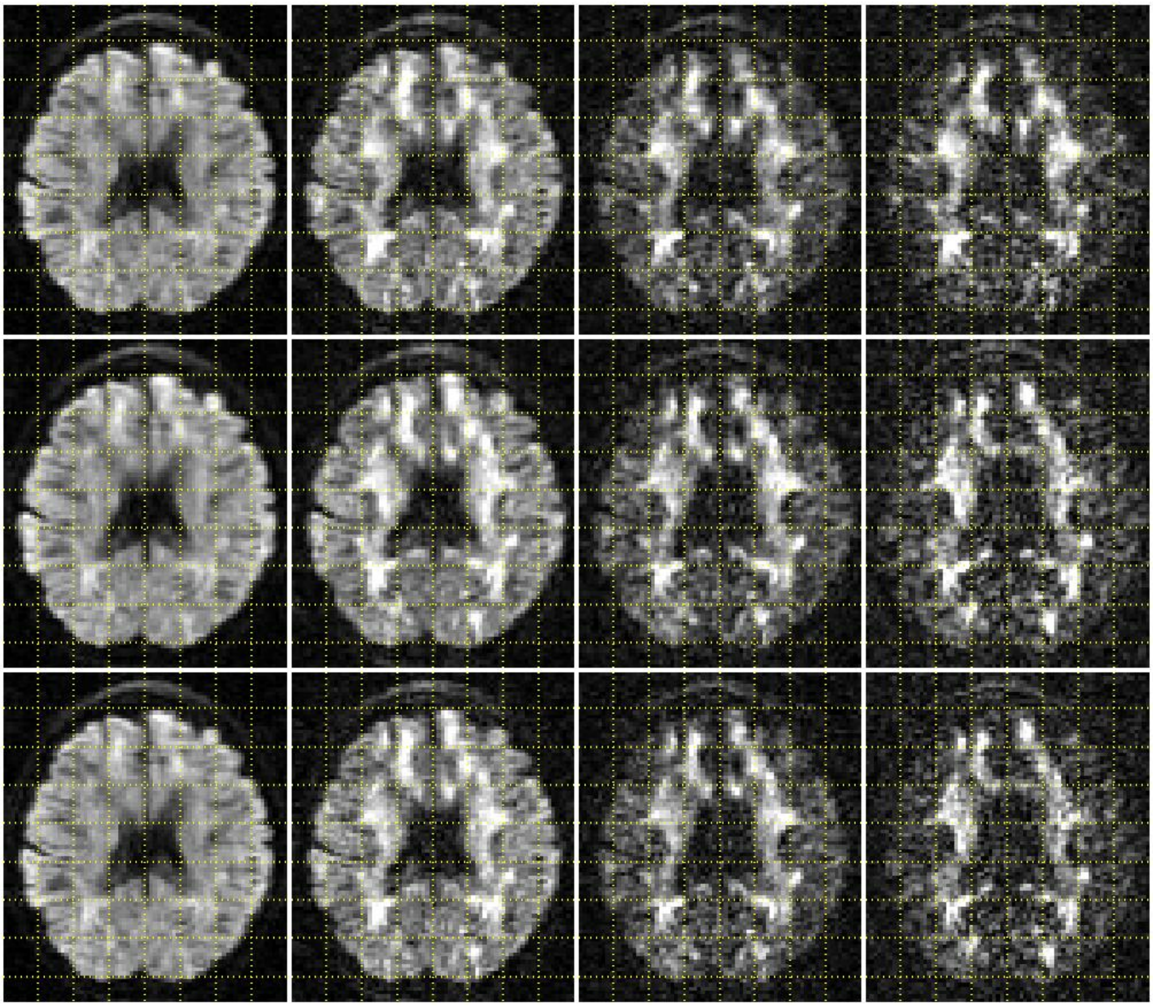

Figure C3: N.B. This is a still from the attached movie movie_old_HCP.gif. The columns show data acquired with, from left to right, b-values of 1500, 3000, 5000 and 7000. The bottom row shows data corrected only for susceptibility, the middle row shows data additionally corrected using `eddy_correct` and the top row after correction with `eddy`. Data set $\mathcal{C}$ was used for this movie.

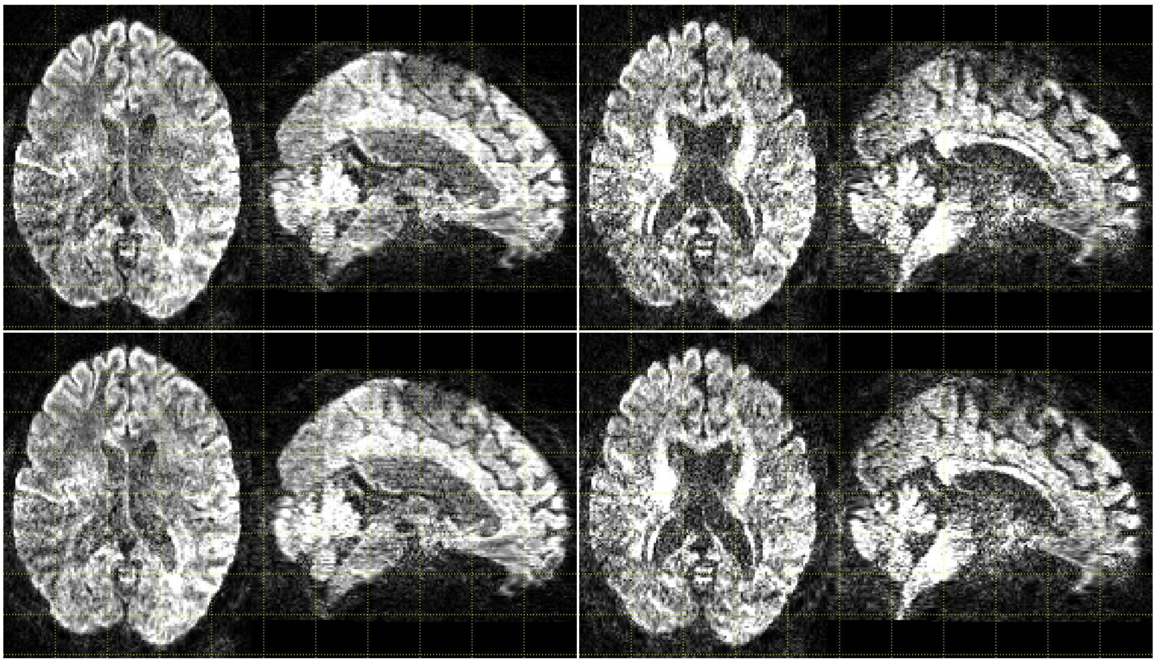

Figure C4: N.B. This is a still from the attached movie movie_7T.gif. The left hand side of the movie shows the HCP 7T data acquired with a b-value of 1000 and the right hand side with a b-value of 2000. The bottom row shows data corrected only for susceptibility and the top row after correction with `eddy`. Data set $\mathcal{E}$ was used for this movie.
